# SM-COLSARSPROT: Highly Immunogenic Supramutational Synthetic Peptides Covering the World’s Population

**DOI:** 10.3389/fimmu.2022.859905

**Published:** 2022-05-25

**Authors:** Manuel A. Patarroyo, Manuel E. Patarroyo, Laura Pabón, Martha P. Alba, Adriana Bermudez, María Teresa Rugeles, Diana Díaz-Arevalo, Wildeman Zapata-Builes, María Isabel Zapata, César Reyes, Carlos F. Suarez, William Agudelo, Carolina López, Jorge Aza-Conde, Miguel Melo, Luis Escamilla, Jairo Oviedo, Fanny Guzmán, Yolanda Silva, Martha Forero, Lizdany Flórez-Álvarez, Wbeimar Aguilar-Jimenez, Armando Moreno-Vranich, Jason Garry, Catalina Avendaño

**Affiliations:** ^1^ Grupos: Síntesis Química, Resonancia Magnética Nuclear y Cálculo Estructural, Biología Molecular e Inmunología e Inmuno-Química, Fundación Instituto de Inmunología de Colombia (FIDIC), Bogotá, Colombia; ^2^ Grupo Inmunovirología, Facultad de Medicina, Universidad de Antioquia UdeA, Medellín, Colombia; ^3^ Núcleo de Biotecnología, Pontificia U. Católica de Valparaíso, Valparaíso, Chile; ^4^ Facultad de Ciencias Agropecualrias, Universidad de Ciencias Aplicadas y Ambientales (UDCA), Bogotá, Colombia

**Keywords:** modified synthetic peptides, SARS-CoV-2, mutational variants, multiepitope, supramutational, worldwide coverage

## Abstract

Fifty ~20–amino acid (aa)–long peptides were selected from functionally relevant SARS-CoV-2 S, M, and E proteins for trial **B-21** and another 53 common ones, plus some new ones derived from the virus’ main genetic variants for complementary trial **C-21**. Peptide selection was based on tremendous SARS-CoV-2 genetic variability for analysing them concerning vast human immunogenetic polymorphism for developing the first supramutational, Colombian SARS-protection (SM-COLSARSPROT), peptide mixture. Specific physicochemical rules were followed, i.e., aa predilection for polyproline type II left-handed (PPII_L_) formation, replacing β-branched, aromatic aa, short-chain backbone H-bond-forming residues, π-π interactions (n→π* and π-CH), aa interaction with π systems, and molecular fragments able to interact with them, disrupting PPII_L_ propensity formation. All these modified structures had PPII_L_ formation propensity to enable target peptide interaction with human leukocyte antigen-DRβ1* (HLA-DRβ1*) molecules to mediate antigen presentation and induce an appropriate immune response. Such modified peptides were designed for human use; however, they induced high antibody titres against S, M, and E parental mutant peptides and neutralising antibodies when suitably modified and chemically synthesised for immunising 61 major histocompatibility complex class II (MHCII) DNA genotyped *Aotus* monkeys (matched with their corresponding HLA-DRβ1* molecules), predicted to cover 77.5% to 83.1% of the world’s population. Such chemically synthesised peptide mixture represents an extremely pure, stable, reliable, and cheap vaccine for COVID-19 pandemic control, providing a new approach for a logical, rational, and soundly established methodology for other vaccine development.

## Introduction

Two years after the SARS-CoV-2 virus was discovered, 6.1 million deaths had been recorded worldwide and 460 million people had become infected (1), leading to severe, universal public health and socio-economic problems.

Around 280 biologically derived vaccine candidates currently in development (some in phase IV clinical trials) are grouped by platform as being traditional (inactivated/live virus vaccines), recently licensed (recombinant protein/virus vectored) and/or never having been licensed (mRNA/DNA vaccines) (2); however, none are chemically synthesised. Data regarding the first synthetic peptide mixture, COLSARSPROT (3), have been published recently.

**Figure 1:**
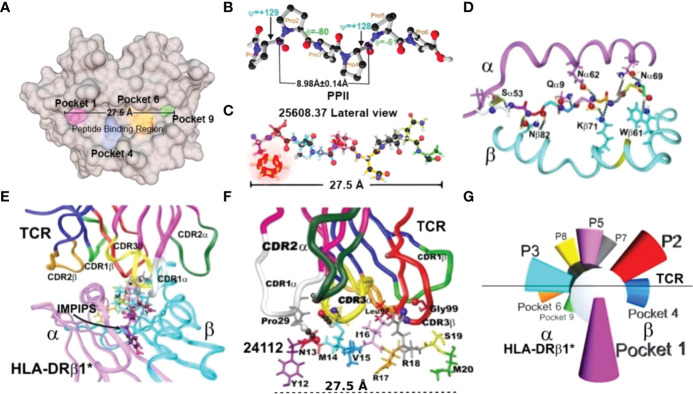
Immune activation system. **(A)** Top view of the MHCII peptide binding region (PBR), highlighting Pockets 1 (fuchsia), 4 (blue), 6 (orange), and 9 (green) (6, 7). **(B)** Top view of PPII_L_ 3D structure showing φ (−80°) and ψ (+129°) angles and 8.98 ± 0.14 Å distance between every three residues, determined by X-ray crystallography (8). **(C)** ^1^H-NMR-determined malaria peptide **25608** 3D structure showing the 27.5 Å distance. The φ: −74.5° ± 14.0° and ψ: +121.8° ± 18.0° angles in this highly immunogenic peptide fitted perfectly into the HLA-DRβ1*0401 PBR (9). α- and β-chain aa backbone atoms are shown, emphasising aa **side chains** establishing H-bonds (silver dots) with malarial peptide **25608 backbone** atoms (9). **(D)** Front view of the HLA-DRβ1*-peptide complex, showing α-chain (deep pink) and β-chain (deep blue). **(E)** View of the HLA-DRβ1*–peptide–TCR complex, showing TRC α-chain (deep pink) and β-chain (deep blue) with complementary determining regions (CDR) side-chain atoms in ribbon: 1α (white), 2α (deep green), 3α (yellow), 3β (red), and 1β (light green) (10). **(F)** Side-view of TCR-CDR establishing H-bonds with peptide aa **side-chain** atoms and 27.5Å distance between the most distant atoms fitting into P1 to P9 (10). **(G)** Diagram giving a front view of the orientation of peptide side chains fitting into the HLA-DRβ1*PBR and contacting the TCR to form the HLA-DRβ1*–peptide–TCR complex.

For more than three and a half decades, we have proposed a minimal subunit-based, multiprotein, multiepitope, chemically synthesised, and structural–functional–immunological approach for dealing with such large-scale infectious disease problems (4, 5). Such approach involved selecting short (20-mer-long) spike (S)–, membrane (M)–, and envelope (E)–derived proteins’ functionally relevant amino acid (aa) sequences, having random coil or β-sheet structures. However, most had to be specifically modified as they were “immunologically silent” [i.e., three-dimensional (3D) structural conformation-related major histocompatibility complex class II molecules (MHCII) [Human Leukocyte Antigen–DR isotype (HLA–DR) in humans] have poor binding ability or lack it, hampering them inducing an immune response] (**Figures 1A**, **D–G**).

COLSARSPROT emerged from our fundamental, thoroughly analysed data, amassed over 35 years’ malaria vaccine research (4, 5); physicochemical rules for modifying and making malaria high activity peptides (HAP) strongly immunogenic and protection (11–13) or neutralising antibody (NAb) inducers against SARS-CoV-2 infection led to the first multiprotein, multiepitope, minimal subunit-based, chemically synthesised, and highly immunogenic peptide mixture against SARS-CoV-2 (3). It was tested in MHCII DNA-genotyped *Aotus* monkeys for use in humans [great similarity (SI) and identity (ID) regarding human HLA-DR immune response genes and molecules] as antigen presentation by a host’s MHCII molecules for appropriate Ab production is the first/most critical step in immune activation. It induced strong immunogenicity in the immunised monkeys (48.25% coverage for 6 weeks and 33.65% for 15 weeks).

Tremendous SARS-CoV-2 genetic variability led expert committees to classify variants having genetic changes predicted or known to affect viral characteristics as “variants of interest (VOI)”, “variants of concern (VOC)”, and variants under monitoring (VUM). These were grouped according to lineage by Greek (α, β, γ, δ, ϵ, μ, ó, etc., ó being recognised as a VOC after this work was finished) and Pango letters (A, B, C, P, R, and Y lineage) (14, 15).

S, M, and E protein variability has made most biologically produced vaccines (>800 S protein–based variants) limited or irrelevant regarding protection-inducing efficacy in some populations (15, 16), i.e., new vaccines must be developed (17).

The aforementioned methodology overcoming the main VOC and VOI mutations and modifying them to fit property into highly polymorphic HLA-DRβ1* molecules led to the SUPRAMUTATIONAL concept and methodology for obtaining the first-generation supramutational peptide mixture (SM-COLSARSPROT). It was predicted to cover 77.5% to 83.2% of the world’s main ethnic groups (average 78.5%) to cope with daunting scientific problems: viral genetic variability and human immunogenetic (HLA-DRβ1* molecules) polymorphism. It induced the desperately needed/sought-after “collective or herd immunity”, close to the 80% coverage recently established by the WHO. Complementary monkey trials **B-21** and **C-21** were designed to solve most physicochemical constraints (i.e., avoid H-bond formation in the aa inside the peptide-binding region (PBR) and avoid residues disrupting PPII_L_ structure) regarding chemically synthesised vaccine development, this manuscript’s *raison d’être*.

## Materials and Methods

### Peptide Synthesis and Characterisation

The solid-phase peptide synthesis (SPPS) described by Merrifield and modified by Houghten (18) was used for peptide synthesis. Briefly, methylbenzhydrylamine hydrochloride (MBHA) resin was swollen with dichloromethane (DCM) and neutralised with 10% triethylamine (TEA) in DCM. Coupling reaction involved activating alpha-L-Boc aa with dicyclohexilcarbodiimide (DCC) and 1-hydroxy-benzotriazole (HOBt) in N,N-dimethylformamide (DMF) for 1 h; coupling reaction verification was followed by the Kaiser test. The resin was then washed and treated with 40% trifluoroacetic acid (TFA) and 0.01% anisole solution in DCM for 30 min to remove t-Boc protecting group. Successive cycles of coupling, deprotection, and neutralisation followed until each peptide’s final sequence was obtained. Peptides were cleaved from the resin by low–high hydrogen fluoride (HF) procedure. Peptides were extracted with TFA, washed with ethyl ether and dried for further characterisation.

The peptides were analysed by reverse-phase high-performance liquid chromatography (RP-HPLC and Chromolith RP-18e column) and by matrix-assisted laser desorption/ionization-time-of-flight (MALDI-TOF) mass spectrometry (MS). Peptides were purified by semi-preparative chromatography. Selected fractions were collected and lyophilised for obtaining pure peptides for monkey and human immunisation.

### Circular Dichroism Spectroscopy

A JASCO J-815 circular dichroism (CD) spectrometer was used for determining peptide CD (JASCO Corp., Tokyo, Japan) in the far ultraviolet range (190–250 nm) at 27°C and 37°C, using 0.1-cm path length quartz cuvettes and 1-nm bandwidth. Each spectrum was recorded as the average of three repeat scans in continuous scanning mode (100 nm/min scanning speed and 2-s response time). Molar ellipticity was calculated for each peptidestock concentration of 2 mg/ml in 1.3 mM phosphate-buffered saline (PBS) with 6.7 mM potassium fluoride (KF) and 30% (v/v) 2,2,2-trifluoroethanol medium. Spectra Manager software (version 2.0) was used for analysing the data and the Contin algorithm and SP37A database were used for deconvolution (19).

### Immunofluorescence Antibody (IFA) Assays

The Universidad de Antioquia’s Immunovirology group produced SARS-CoV-2 (wild-type B.1D/614G, α, β, γ, and δ) variant-infected VERO cell cultures. Once fixed on eight-well slides, they were preserved at 4°C and left to dry at room temperature (RT) to evaluate native state viral protein recognition. They were blocked with 30 µl per well 10% bovine serum albumin for 15 min, washed five times for 5 min with PBS and left to dry at RT. Immunised *Aotus* sera (diluted 1:40 to 1:640 in PBS) was placed on slides at 15 µl per well. They were incubated in a humid chamber for 30 min, washed six times with PBS (5 min each) and left to dry. Fluorescein-5-isothiocyanate (FITC)–conjugated goat immunoglobulin G (IgG) (goat IgG-FITC) against purified *Aotus* IgG (produced in our Institute) was then added at 1:100 dilution in PBS, 1:100 Evans blue; plates were incubated in a humid and dark chamber for 30 min and washed six times with PBS and left to dry. A drop of 50% glycerol in saline solution was added between the slides and microarray coverslips before observing them by fluorescence microscope (100× oil immersion objective lens).

### Enzyme-Linked Immunosorbent Assays

Enzyme-linked immunosorbent assay (ELISA) involved 96-well ELISA plates (F16 MAXISORP plates) was covered with 100 µl per well test of native unmodified parental peptide at 10 µg/ml in PBS and incubated for 1 h at 37°C and overnight at 4°C. The sensitised plates were incubated at 37°C for 1 h, washed five times with PBS, 0.5% Tween-20, followed by a final wash with distilled water. They were blocked with 200 µl per well blocking solution (PBS, 0.5% Tween-20, 5% non-fat milk) and incubated at 37°C for 1 h. Then, 100 µl per well immunised *Aotus* sera were placed in wells at 1:100 dilution, incubated at 37°C for 1 h, and washed again; 100 µl per well of goat anti-*Aotus* IgG peroxidase conjugate was then added at 1:1,000 dilution in blocking solution. Plates were incubated at 37°C for 1 h and washed five times, and 100 µl per well developing solution was added (TMB substrate peroxidase and hydrogen peroxide: 1:1 ratio). Microplates were read (LabSystems Multiskan MS) 30 min later at 620 optical density (OD).

### Neutralising Antibody Assays

The Universidad de Antioquia’s Immunology Group’s BSL3 laboratory used Vero E6 cells for a 50% ± 3% plaque reduction neutralisation test (PRNT50), using monkey sera having the B.1 D614G variant. Briefly, Vero E6 cells (1.1 × 10^5^ cells per well) were seeded onto 24-well tissue culture plates the day before infection. The next day, 80 plaque forming units (PFU) of SARS-CoV-2 were incubated with or without serially diluted *Aotus* heat-inactivated sera (56°C, 30 min) at 200-μl volume in microcentrifuge tubes for 60 min at 37°C in 5% CO_2_. The mixtures were then added to Vero E6 monolayers and incubated at 37°C for 60 min; the inoculum was removed, 1 ml of semisolid medium added (1.5% carboxymethyl cellulose, 2% FBS, 1% streptomycin, and DMEM).

Mixtures were cultured at 37°C for 72 h; semisolid medium was removed and monolayers were washed twice with PBS, fixed and stained with 4% formaldehyde/1% crystal violet for 30 min, and washed twice with PBS. A 50% ± 3% reduction in plaque count (PRNT50) was used as neutralising end-point. Viral control (VC) in the absence of serum and a serum control without SARS-CoV-2 were included in the tests (20, 21).

### Blood Samples and PBMC Isolation for Cellular Immunity Studies

Venous blood was collected in ACD vacutainers (Becton Dickinson, United Kingdom) before vaccination and 6 weeks after the second immunisation dose. Ficoll-Paque PLUS sterile density media (GE Healthcare, United States) was used for isolating peripheral blood mononuclear cells (PBMCs) from whole blood.

### T-Cell Proliferation Assays

*Aotus* **B-21** and **C-21** PBMC were seeded at 10^5^ cells per well density. Group I was stimulated with a 50-peptide mixture (10 μg/mL) of S, M, and E proteins and Group II with modified peptides derived from previously reported T-cell activating sequences: CD4^+^ T cells with native parental peptides **42992** (152–171), **43016** (369–388), **42964** (402–421), **42956** (446–464), **42962** (460–479), **42948** (485–500), and **43036** (1,110–1,129). CD8^+^ T cells were used with **43002** (386–405), **42970** (977–996), **42954** (1,027–1,046), **43040** (1,027–1,046), and **42960** (1,176–1,194) (10 μg/ml). The medium was used as negative control and phorbol myristate acetate (100 ng/ml) and ionomycin (400 ng/ml) as positive control for both groups (20).

All stimuli were run in triplicate and all treatments were analysed after 5-day coculture. A FACS Canto II cytometer (BD Sciences) was used for reading positive CD4+/CD45RO+ and CD8+/CD45RO+ cell percentages; FlowJo V10 (Ashland, Oregon, USA) was used for data analysis. A BD cytometric bead array (CBA) NHP Th1/Th2 cytokine kit (BD Biosciences) was used for cytokine analysis. Mann–Whitney and Student’s tests were used for assessing statistically significant differences between groups.

### Genotyping Non-Human Primate MHC Class II Genes

#### Collecting Samples, Extracting DNA, and Determining Species

Peripheral blood (PB) samples were taken from 61 *Aotus* sp. monkeys kept at Fundación Instituto de Inmunología de Colombia’s (FIDIC) primate station in Leticia (Colombia’s Amazonas department). A Wizard Genomic DNA Purification Kit (Promega) was used for isolating *Aotus* genomic DNA (gDNA) from 300 µl of PB samples, according to the manufacturer’s instructions. The D-loop gene [part of mitochondrial DNA (mtDNA)] was amplified for determining the species (22).

### Amplicon-Based Sequencing

*Aotus* MHC DRβ class II exon 2 was amplified by PCR; primers were designed from previously reported *A. nancymaae* and *A. vociferans* allele lineages using intron 1 and intron 3 sequences (23).

A 317–base pair (bp) MHCII DRB gene exon 2 fragment was amplified. The Barcosel web tool was used for selecting an optimal barcode set for high-throughput sequencing for assigning each primer a 6-bp-long molecular identifier (24, 25).

Phusion Hot Start II High-Fidelity PCR Master Mix was used for amplification, using the touchdown PCR technique. A Wizard SV Gel and PCR Clean-Up System Kit (Promega) was used for purifying PCR products, following the manufacturer’s recommendations. A NanoDrop 2000 full-spectrum UV-Vis spectrophotometer was used for quantifying products that were sent for Illumina NovaSeq 6000 sequencing.

### Genotyping by Amplicon In-Depth Sequencing

Sequence data were processed using Amplicon Sequencing Analysis Tools (AmpliSAT) suite; AmpliCLEAN was used for removing low quality (<30 Phred) reads and non-amplicon sequences. AmpliCHECK was used for analysing MHC DRB exon II using a 1% base substitution error rate, a 0.001% indel error rate (base insertion or elimination) and 3% minimum per-amplicon frequency (PAF) (26). The maximum number of alleles per amplicon was set and reading depth was limited to ~80,000 reads for all amplicons analysed by AmpliSAS. Minimum read depth per amplicon was set at 100 (all amplicons having less than 100 reads were excluded); the software identified and removed chimeras (variants arising from parenteral sequences in the same amplicon). The ACACIA pipeline was used with default parameters and 0.03 proportion threshold (low-poor) for allele calling (code available under https://gitlab.com/psc_santos/ACACIA) (27). Sequences were deposited in GenBank (**Supplementary Table S1** gives accession numbers).

### MHC-DR Peptide-Binding Prediction, Binding Profiles, and Potential Epitope Population Coverage

The Allele Frequency Net Database (AFND) was used for data mining and choosing HLA-DRβ1* alleles (24) occurring with greater than 1% frequency in the database-determined ethnic groups (69 alleles). Previous reports were used for *Aotus* MHC- DRβ and current typing for establishing allele frequency (28, 29).

NetMHCIIpan-4.1 was used for evaluating and selecting proposed peptides’ ability to bind to selected HLA-DRβ1* alleles, i.e., having strong BA and/or presentation ability (≤2.5 percentile range), being non-binders and/or lacking presentation ability (≥10). The server was also used for calculating *Aotus* MHC-DR BA and sequence typing. A set of 200,000 non-redundant UnitProtKB/Swiss-Prot UniProt-derived peptides (13- to 21-aa-long) (30) was used for calculating the predicted peptides’ percentile rank distribution. Seq2Logo (31) was used for constructing peptide binding motifs and scoring matrices for each allele based on stringent binding cores (<2.5 percentile rank). The Kullback–Leiber approach from the previous step was adopted for calculating an SI index for comparing each allele’s pair, using MS eluted ligand (EL) scoring matrices, defined as Pearson distance [centre: 1 − corr(x,y)] with min-max normalisation.

The set of epitopes’ potential coverage resulted from the sum of the alleles’ frequencies in the target human population for which each designed peptide had significant affinity. The frequency of the 69 previously selected HLA-DRβ1* alleles calculated from the AFND (24) was used for estimating world population coverage.

The SI, based on MHC-peptide affinity predictions, determined strong similarity between selected HLA-DR and genotyped *Aotus* MHC-DR (average 83.7 ± 8.5).

### Quantification and Statistical Analysis

ELISA, IFA, and NAb tests were performed several times with at least two independent preparations of each sample.

### Experimental Model and Subject Details

#### Animals and Immunisation Procedures

Colombian environmental agency (CORPOAMAZONIA)–authorised biomedical research involved using 63 wild-caught *Aotus* monkeys from the Amazon jungle [permission granted since 1990, the last version coded 0632 and 0042/2010 was renewed on April 2, 2020 (resolution 0366)]. The monkeys were kept in FIDIC’s field station in Leticia, Colombia (Amazonas department), looked after by expert primate veterinarians and workers and supervised weekly by expert biologists and veterinarians from the local environmental authorities and ethics committees.

They were subcutaneously immunised at the Primate Centre with 3 mg per dose peptide mixture in equimolecular concentrations. The first immunisation (day 0) involved the mixture being dissolved in 200 microliters sterile double-distilled water (ddH_2_O) emulsified with an equal volume of Freund’s complete adjuvant (FCA). The second immunisation (day 20 in **B-21** and day 30 for **C-21** trials) involved the same amount of SM-COLSARSPROT mixture emulsified with incomplete Freund’s adjuvant in equal volumes. Bleeding (1.5 ml of PB) for IFA and NAb immunological analysis using individual native peptides and ELISA assays were drawn in pre-immune sera, 20 (II_20_) and 160 (II_160_) days after the second immunisation for **B-21** and 30 (II_30_) and 75 (II_105_) days for **C-21**.

One hundred eighty days represented the longest immunisation follow-up time for any SARS-CoV-2 vaccine by the time this trial was finished. The 61 immunised monkeys in the **B-21** and **C-21** trials were DNA genotyped regarding their class II region to match human class II HLA-DRβ1* genes during the time the experiments lasted, because *Aotus* immunogenetic typing data had to be extrapolated to humans.

**B-21** involved monkeys being maintained for an additional 4 months (local authority-authorised) to determine immune response duration. All were then released back into the jungle close to their capture sites following agreement with the pertinent environmental authorities; environmental authority officials (CORPOAMAZONIA) and some members of the ethics’ committees supervised these events.

### Peptide Selection

Our previous studies on malaria peptide structure has provided the basis for suggesting that chemically synthesised peptide structure (according to native protein aa sequence) means that they maintain their secondary structure, whether this be α-helix, β-turn or random coil. This was assumed for SARS-CoV-2 synthetic peptide production.

Peptides selected as vaccine candidates had to comply with two, well-established, fundamental requirements:

First, they had to have been 20-aa-long and preferably had random coil or β-sheet secondary structure in the native protein. UCSF Chimera (32) was used for verifying such structures, i.e., the phi and psi angles in the 3D structure of already reported S, E, and M proteins [protein data bank (PDB): S protein: 6X6P, 6VXX, and 6VYB; E protein: 5X29 (33–36)]. Whereas the algorithms included in Swiss Institute of Bioinformatics (SIB) Resource Portal’s Expasy Tools (37) were used for predicting secondary structure for M and ORF9c proteins. Regions containing random coils or beta sheets were selected.

Second, it was preferred that most fragments selected by the secondary structure criteria fulfilled relevant biological functions in the target proteins. Selected S protein peptides were involved in angiotensin-converting enzyme 2 (ACE2) binding in the receptor binding domain (RBD) (38) and protein interaction with linoleic acid (LA) (39), cleavage sites S1 and S2’, membrane fusion sequence (40), linkers, heptad regions 1 and 2 (HR1 and 2) (41), and the polybasic binding site (PBBS). Selected E- and M-derived peptides were also involved in biological functions, e.g., ion and nutrient transport in the E protein and the PDZ binding motif (PBM) (42).

However, some helical fragments were chosen, because they fulfilled relevant functions in the protein, for example, protein fusion to target cell.

The selected peptides were called native or parental peptides; these were modified in some aa, which were critical regarding peptide binding to the MHCII-PBR [thereby obtaining modified peptides mHAPs], according to previously reported rules and principles, i.e., changing aa polarity whilst maintaining volume and mass) for obtaining immunogenic peptides, which were capable of inducing protection (11, 13, 43).

It should be mentioned that the S protein has an antibody-dependent-enhancement region (ADE) located between aa 597 and 630; it is known that Ab-based therapies and vaccines involve a risk of increasing COVID-19 severity through this Ab-dependent enhancement (ADE) mechanism (44), peptides in this aa region were thus not selected.

## Results

### Structural-Functional Analysis for Peptide Selection

Parental/native aa sequences were selected for modifying and producing SM-COLSARSPROT. They had to have random coil or β-sheet structures, perform relevant functions or mediate fundamental inter- or intra-molecular interactions, and have little genetic variability (some mutated peptides were exempt from the last two requirements due to their relevant invasion-related role).

**Figure 2:**
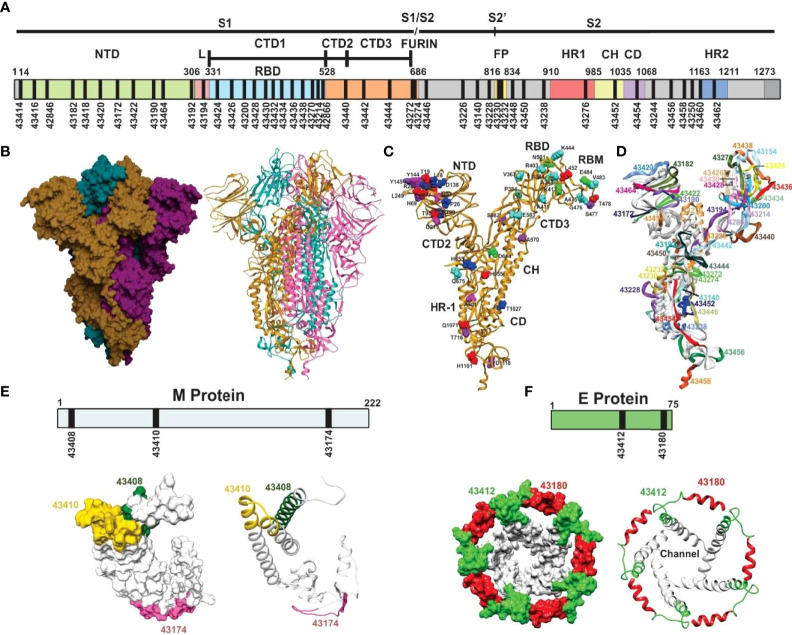
Schematic representation of SARS-CoV-2 S protein. **(A)** Schematic representation of the SARS-CoV-2 S protein. The S1 subunit containing the N-terminal domain (NTD), connecting loops in C-terminal domains CTD1, 2, and 3, mediating hinge up or down CTD1 rotation, exposing the receptor site for interaction with the ACE2 protein, the cell tropism-mediating, host range, and transmission receptor binding domain (RBD) and the S1/S2 furin site for S cleavage into S1 and S2 subunits. The S2 subunit contains the fusion peptide (FP) hydrophobic fusion-peptide proximal region (FPPR), the heptad-repeat regions (HR1 and HR2) with their connecting domains (CD) enabling six helix bundle fusion core conformation to mediate host cell invasion. **(B)** Trimer S protein Cryo-EM structure [surface and ribbon representation (33, 34), each protomer shown by a different colour]. **(C)** S protein protomer ribbon diagram showing VOC mutant residue locations: alpha (green), beta (fuchsia), gamma (dark blue), delta (red), and common (orange) and VOIs in aquamarine (epsilon, eta, and iota). **(D)** S protein protomer backbone structure (ribbon), showing the location of selected peptides to be modified for SM*-*COLSARSPROT, numbered according our Institute’s serial numbers (**Figures 5**, **6**). **(E)** Predicted M protein structure (36) (surface and ribbon) with selected regions (numbers are our serial numbers). **(F)** ^1^H-NMR determined SARS-E protein structure (35) (surface and ribbon) with selected regions (numbers are our serial numbers), showing the ion channel.

**Figure 3:**
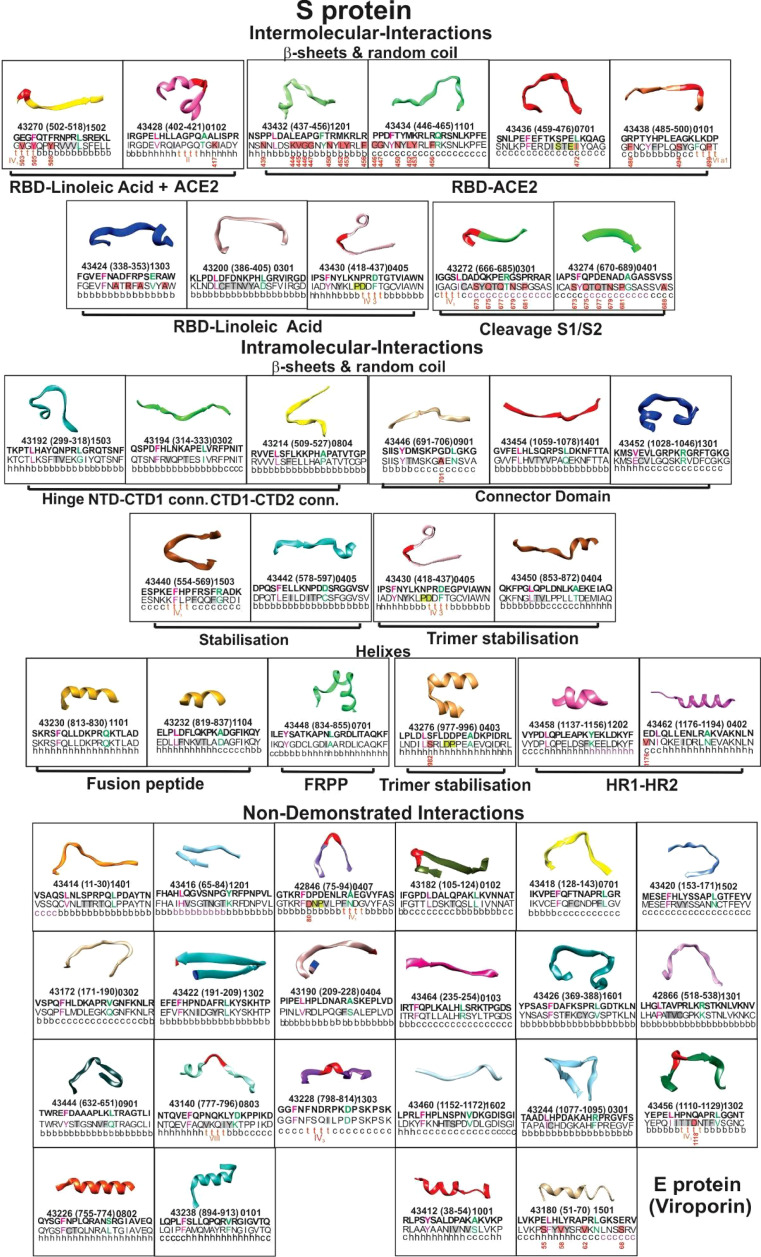
Selected peptides’ structure. The boxes show ribbon representations of selected S (PDB code: 6X6P, 6VXX, and 6VYB) and E (PDB code: 5X29) peptides, according to their 3D structure, classified as β-sheet, random coil, α-helixes (on top), and functional activities (bottom in bold letters). Inside, the Institute’s peptide numbers, their location in the aa sequence (in parenthesis) and HLA-DRβ1* allele they bind to. Modified peptides’ aa sequences above in bold letters; native peptides’ aa sequences below, with secondary structure. Red bold and green letters show residues fitting into HLA-DRβ1* P1 to P9. Red shading shows aa having a high mutation rate with position number in the aa sequence; grey shading shows aa having low PPII_L_ propensity (Aro, β-branched aa). Their functional activities are shown in bold letters outside the boxes.

S (PDB code: 6X6P, 6VXX, and 6VYB) (33, 34), E (PDB code: 5X29) (35), and M protein (36) predicted structures were used for structural selection (**Figure 2**). Trial B-21 involved 44 S protein peptides, 3 M protein-derived ones, 2 E protein peptides, and 1 ORF9c protein-derived peptide for determining the 50 modified peptides’ immunogenicity and immunity duration (180 days). Trial C-21 involved 53 molecules: 47 S protein, 38 β-sheet, and random coil or β-turn structures; 27 had relevant functions, plus another 20 for which no functional activity has yet been demonstrated. Nine of the 47 S-derived peptides had α-helix structures (7 being very relevant during invasion). Both E protein peptides had α-helixes (**Figure 3**). Eleven functionally relevant peptides identified in S protein’s S1 region and cleavage sites S1/S2 were directly involved in intermolecular interactions with angiotensin converting enzyme 2 (ACE2) (38) and linoleic acid (LA) (39) host cell attachment and invasion-mediating receptors (**Figure 3**).

Hinge and trimer stabilisation regions (45) previously recognised by other groups (46) were identified and modified in the ten peptides involved in fundamental S protein peptide NTD1-CTD1 and CTD1-CTD2 intra-molecular interactions. Two were membrane fusion peptide (FP) components, one was involved in fusion peptide proximal region (FPPR) formation (pH-dependent structure and function) (47), two in HR1-HR2 intersection, involved in six-helix bundle formation (40) and one in trimer stabilisation (**Figure 3**). E protein (viroporin) peptides were directly involved in ion and nutrient transfer-related channel formation (**Figures 2F**, **3**); two of three M protein peptides (lacking 3D structure determination) were predicted to have random coil structures theoretically interacting with other SARS-CoV-2 proteins: one α-helix (**Figure 2E**) (36) and one ORF9c protein-derived peptide.

### Native Peptides’ Immunogenetic Characteristics

**Figure 4:**
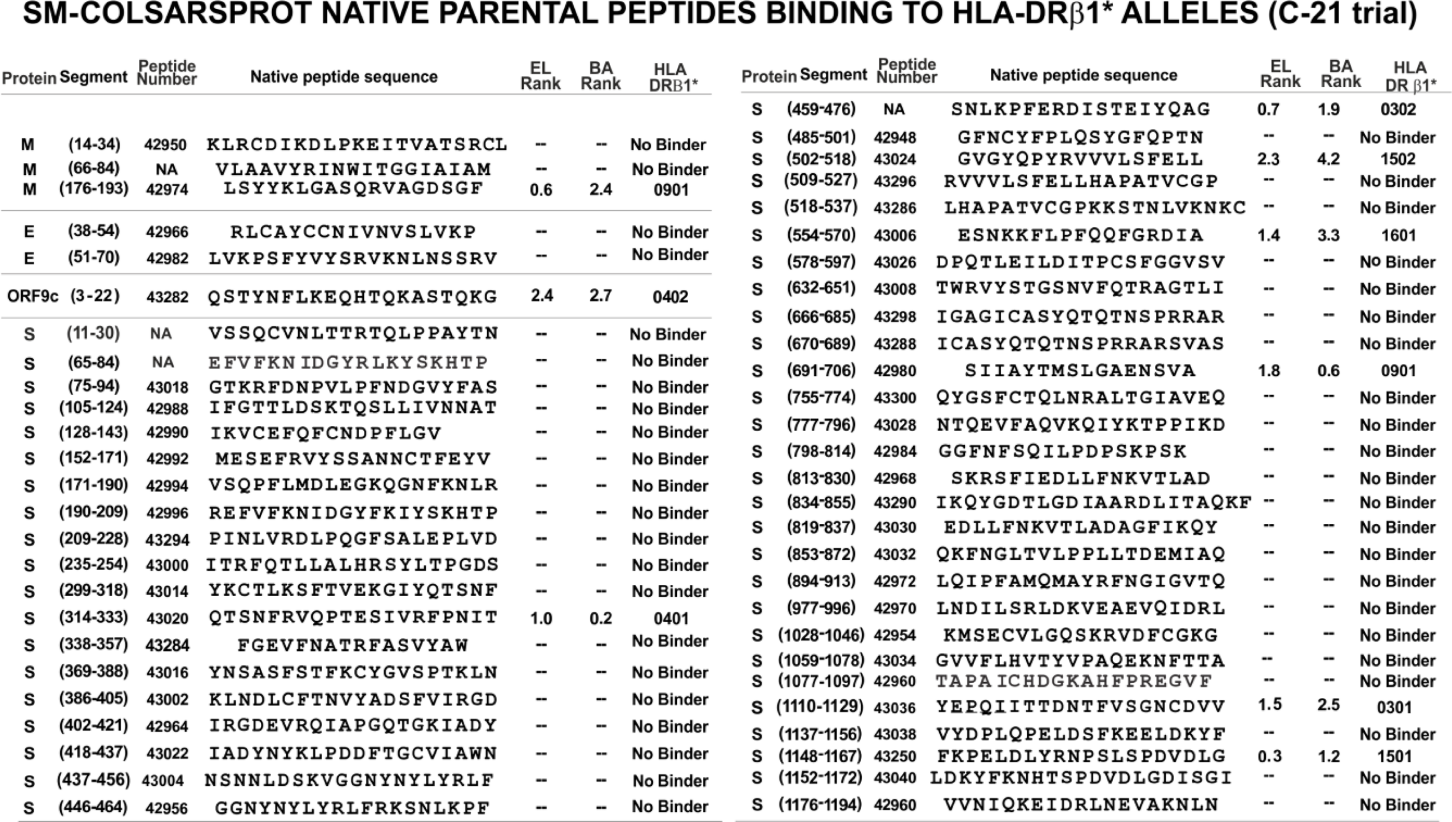
Native selected peptides, based on their structural and functional characteristics. The aa sequences reported for B.1/D614G strain were analysed for their elution (EL) to HLA-DRβ1* purified molecules and homozygous cell binding activity (BA): NetMHCIIpan-4.1 server-predicted peptide *b*inding: <2.5 percentile (i.e., threshold level) and core probability close to 1.0. They did not bind, even at >10 percentile rank.

NetMHCpanII 3.2/4.1 (48, 49) strikingly predicted that 44/53 (83%) SM-COLSARSPROT native parental peptides in C-21 lacked HLA-DRβ1* and/or Aona-DRB allele-binding capability [elution (EL) or binding activity (BA)] (**Figure 4**). This partially explained their poor/weak immunogenicity and/or antigenicity (50); the remaining nine peptides (17%) bound to a limited amount of alleles due to inappropriate TCR-contacting residues, suggesting the need for further structural modification to make them highly immunogenic and NAb-inducers.

**Figure 5:**
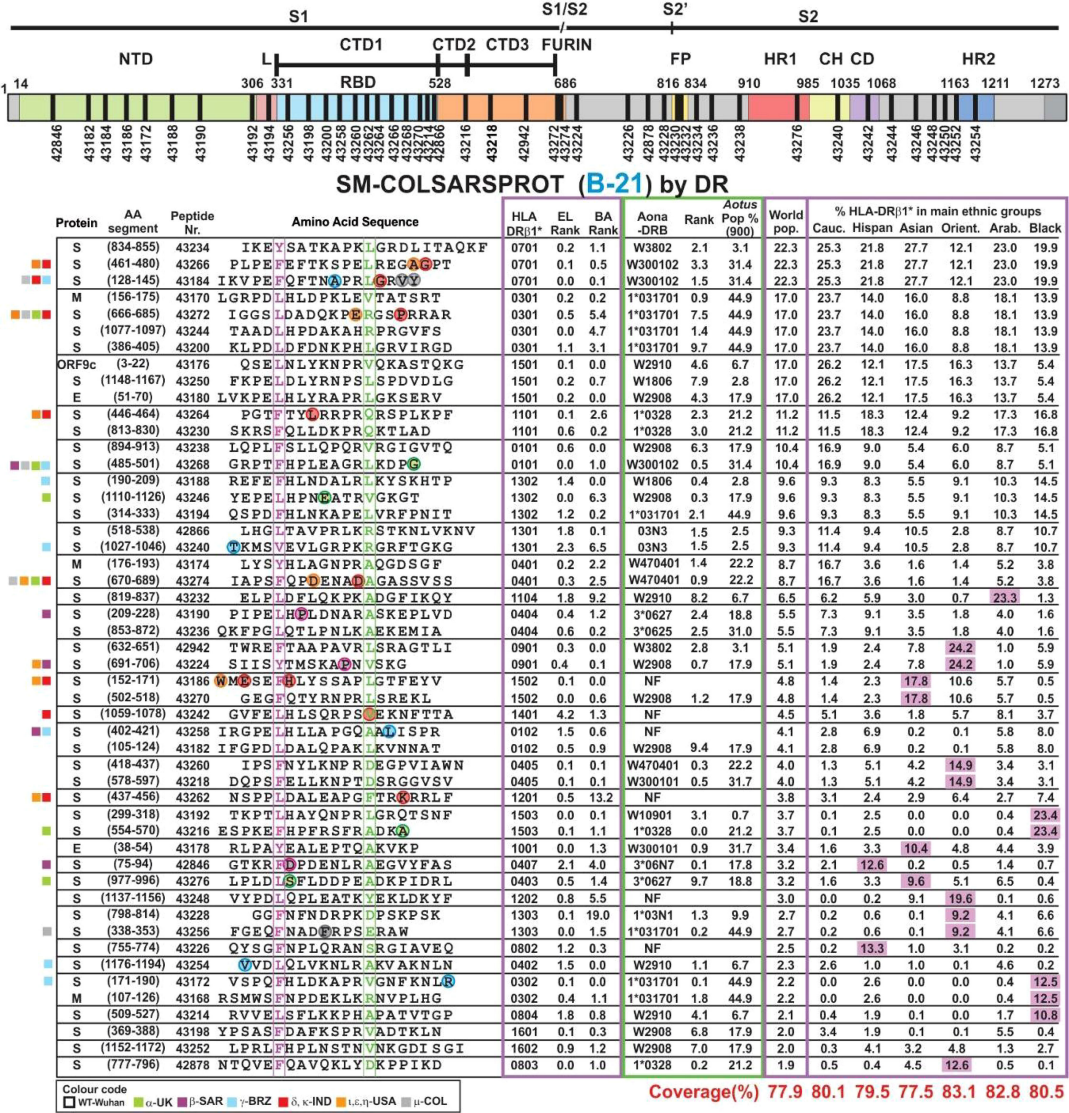
SM-COLSARSPROT modified peptide aa sequences in B-21. Schematic representation of the S protein showing the modified peptides’ location. Columns grouped according to the most frequently occurring HLA-DRβ1* alleles. Proteins: S, M, and E; aa segment, numbers in parenthesis show location in the aa sequence; peptide number: FIDIC serial number for modified peptides; their aa sequence boxed and highlighted in fuchsia and green residues fitting into HLA-DRβ1* P1 and P9. Alpha mutant residues enclosed in green, beta in fuchsia, gamma in blue, and delta in red circles replaced in these peptides. Most frequently occurring HLA-DRβ1* alleles in the world’s population with their rank according to EL and BA binding ability. Aona-DRB alleles ranked according to EL and their frequency in the *Aotus* population. World population frequency (%) and these alleles’ presence (%) in the main ethnic groups and Colombians. SM-COLSARSPROT potential world coverage and for the main ethnic groups at the bottom, highlighted in red. Fuchsia: alleles >3× frequency in target population. On the left, coloured squares according to VOC and VOI colour code.

**Figure 6:**
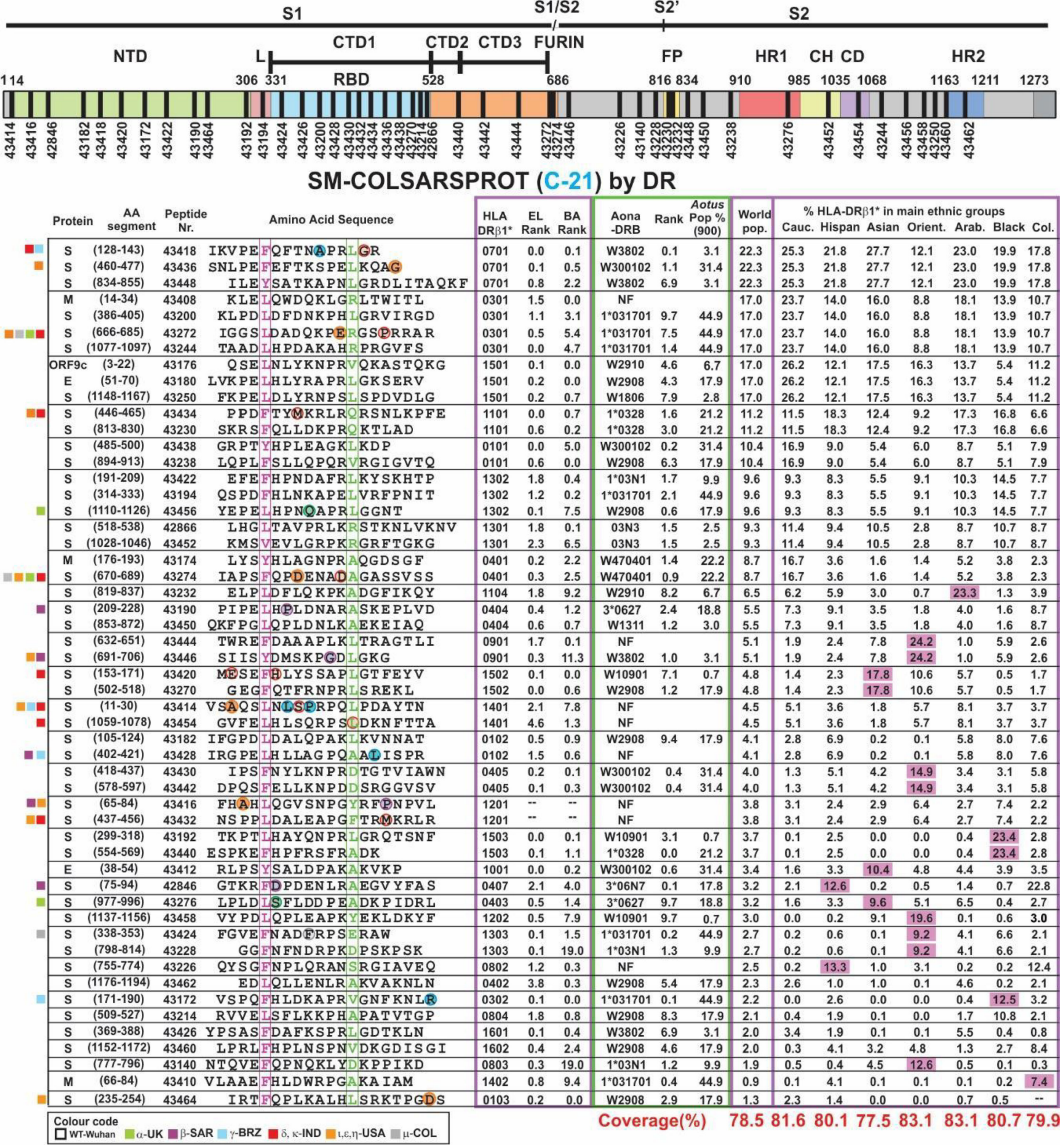
SM-COLSARSPROT modified peptide aa sequences C21. The same as **Figure 3**, but using different peptide numbers representing new modifications in aa sequences and new peptides.

**Figure 7:**
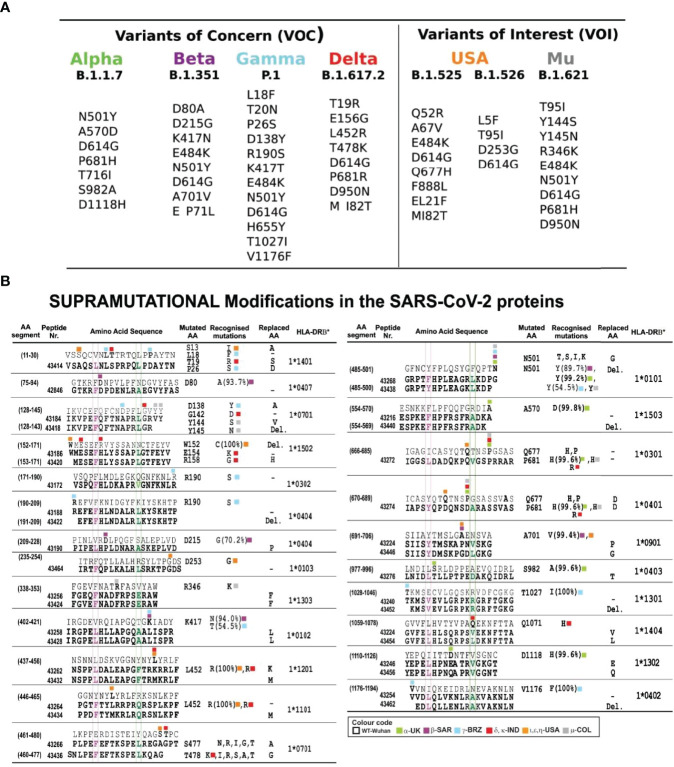
Most frequently occurring mutant residues in VOC and VOI. **(A)** VOC mutations: α, β, γ, and δ and VOI USA and μ from www.cov-lineages.org. **(B)** FIDIC’s serial peptide numbers (grouped according to exclusive or common presence). Numbers in parenthesis show their location in the aa sequence; above, native parental aa sequence, below (bold), modified peptides. Residues fitting into the HLA-DRβ1* P1-P9 boxed in fuchsia and green as reference and modified peptides with residues boxed, coloured according to the variants’ colour code in bold. The mutated aa column shows mutant residue variants, where first identified and their frequency in %; the last column shows the HLA-DRβ1* allele they bound to after modification. Colours show different VOC, VOI, and VUM variants. Native peptides did not bind HLA-DRβ1* molecules or had low BA (**Figure 4**).

**Figure 8:**
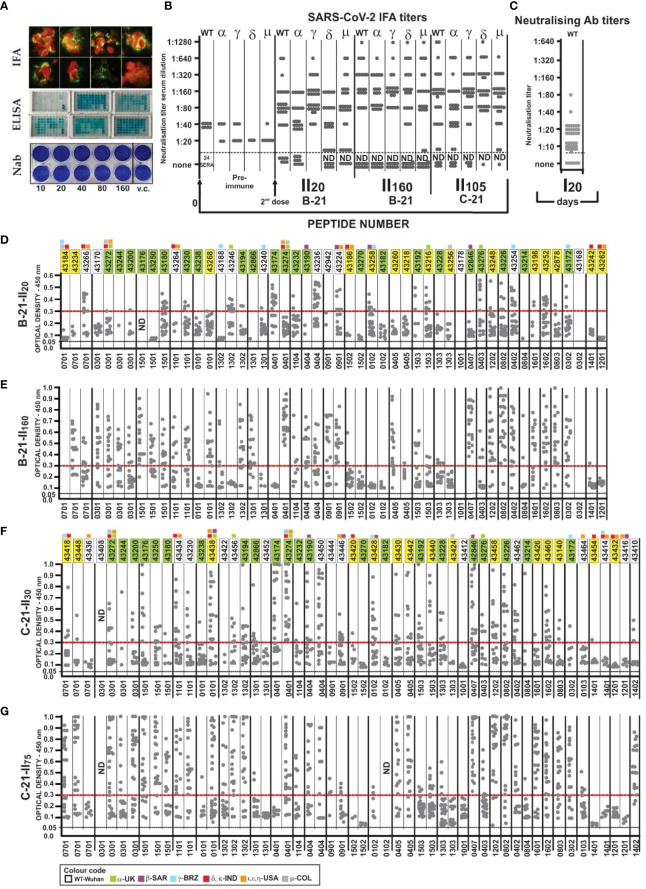
Immunological results. **(A)** IFA reactivity, *Aotus* sera (fluorescent green) with SARS-CoV-2 human/Medellin/V A1/2020 strain. ELISA assay, *Aotus* sera recognition of native peptides; NAb titres (sera dilution), viral control (vc). **(B)** *Aotus* pre-immune sera IFA titres 20 (II_20_), 105 (I1_105_), and 160 days (I1_160_) after the second dose in **B-21** and **C-21** in two-fold dilution with VERO cells infected with wild-type (B.1/D614G), Alpha, Beta, Gamma, Delta, and Mu variants. **(C)** NAb titres with wild-type B.1/D614G (WT) with *Aotus* sera. **(D–G)** *Aotus* sera reactivity (ELISA: 1:100 dilution) against unmodified parental peptides at 450-nm OD with **B-21** (II_20_ and II_160_) and **C21** (II_30_ and II_75_) serum samples grouped according to the HLA-DRβ1* to which they bound (the HLA-DRβ1* associated with each peptide is shown below D, E, F, and G). The first region containing 22 peptides common to **B-21** and **C-21**, 15 having aa sequence variations (1-3 aa) and 13/50 new ones in **B21** and 16/53 in **C21** (colourless), to include new VOC variations, OD 0.05 baseline and above 0.30 strong reactivity.

Seven prior trials (A-20 to F-20 and A-21) were designed to determine the modifications needed to make them highly immunogenic, proline’s role in their conformation, selecting the most immunogenic ones, the amount of peptides to cover >80% of the world’s population and the use of Al(OH)_3_ as adjuvant. This involved 30–50 modified peptides, each used in ~30 *Aotus* monkeys per trial. B-21 involved 50 modified peptides in 32 *Aotus* monkeys (**Figure 5**), C-21 involved 53 peptides in 30 monkeys (**Figure 6**). **Figure 7** shows these peptides’ supramutational modifications to make them highly immunogenic. **Figures 8D–G** list peptides common to B-21 and C-21 (green), 1–3 aa differences between them (yellow), some having newer modifications (colourless) due to residues having VOC and VOI mutations (9, 11–13, 43, 51). Small coloured squares in **Figures 5**–**8** represent peptides in which mutations were replaced, according to colour code.

### Physicochemical Principles for SM-COLSARSPROT Modification: PPII_L_ Conformation Propensity

**Figure 9:**
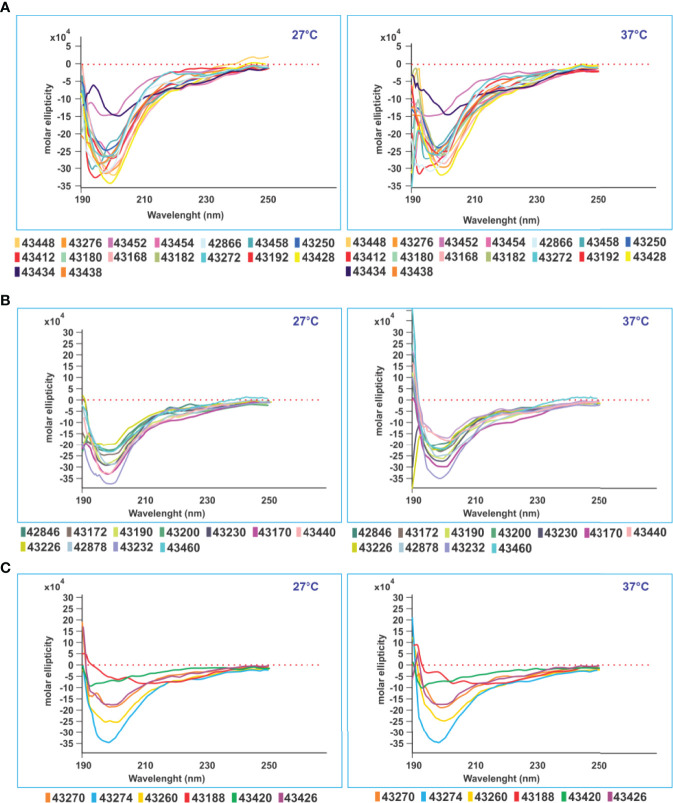
CD structural analysis. CD structural analysis revealed three immunological result-associated structural patterns. **(A)** Sixteen modified peptides having random coil structure with very deep negative ellipticity at 190 nm (starting below 0 at 27° and 37°C) associated with high Ab levels against native parental peptides. **(B)** Eleven modified SM-COLSARSPROT peptides inducing high classical negative ellipticity-associated Ab titres at 190 nm (same parameters). **(C)** Six modified peptides inducing very low Ab levels against native parental peptides had the same negative ellipticity at 190 nm (same parameters), all having random coil secondary structure.

SM-COLSARSPROT peptide design involved determining PPII_L_ conformation propensity by CD spectra (**Figure 9**); selected peptides were modified to ensure PPII_L_ structural propensity (9, 51, 52). Most **C-21** (40/53) native peptides had residues disrupting PPII_L_ structure, i.e., I, V, and T (β-branched aa) and aromatic aa, such as Y, F, and W (**Figure 3**, grey). These native parental peptides also contained polar aa having a short side chain promoting H-bond formation between side chain–backbone (SC-BB) (**Figure 3**, highlighted in green), as well as promoting n→ π*-type interactions (53).

All PPII_L_-disrupting residues had to be replaced by others having similar physicochemical characteristics, such as mass, surface, and volume but opposite polarity (9, 11–13, 43, 51, 52), as well as aa having a strong propensity to form PPII_L_-type (P>>>L>R>A/K>M/D>Q/E>H>>G/N/S/C/T/V/I/F/Y/W) (54). Ile was categorically avoided and replaced by Leu having similar mass and volume, but different conformation and a slight difference regarding polarity (54). **Figures 3**, **7** show native peptide sequences, having the most relevant mutations (highlighted in red) and their locations (modified peptide sequences shown below in bold).

As examples of the role of these PPII_L_ conformation disrupting capacities, non-binding HLA-DRβ1* peptide 42954-derived (1,028–1,046) modified peptides **43240** and **43452** comprising disruptor aa V1031 and V1033 (**Figures 5**, **6**) having strong HLA-DRβ1*1301 and Aona-DRB1*03N3 BA did not induce Ab due to β-branched aa’s negative role (**Figures 8D–G**). **43436** (**NA**:460-477) having F465 bound strongly to HLA-DRβ1* 0701 whilst Aona-DRB W300102 did not induce Ab production. **43186** (42992: 152–171) and **43420** (42992: 153–171) having F157 and Y160 and **43270** (43024: 502–518) having F505 and Y508 designed for strongly binding HLA-DRβ1*1502, Aona-DRB W10901 and W2908 did not induce any Abs. **43424** (43284: 338–353) having strong HLA-DRβ1*1303 and Aona-DRB1*031701 BA was also incapable of inducing Ab production (**Figures 8D–G**). These peptides provided clear examples of β-branched and aromatic (Aro) residues’ negative role when inside the PBR, despite many monkeys having such Aona alleles in the trials.

### Disrupting Critical Side Chain–Backbone H-Bonds to Induce Immunogenicity

Further physicochemical intra-peptide electron interaction-related principles were identified during this study, as most theoretical SC-BB H-bonds were disrupted (whenever possible due to HLA-DRβ1* or Aona-DRB allele restrictions) for a better fit in the MHCII-PBR.

The main SC-BB motifs to be disrupted were NP, PD, PN, DP, TP, and SP, occurring with 14% to 32% frequency in proteins (55, 56). As they are found in native peptides, this makes them candidates for replacement to avoid such H-bond formation so that the structure of the nine aa fitting into the PBR-MHCII is as similar as possible to PPII_L_. This gives a sufficiently stable MHCII-peptide bimolecular complex to ensure TCR recognition, establishing the MHCII–peptide–TCR trimolecular complex, thus triggering an immune response.

SC-BB interactions’ negative role was better observed when peptides were modified to ensure HLA-DRβ1* BA (DRβ1*0701, 1201, 1401). Such compulsory requirement insisted on small polar residues (N, T, D, and S) in Pockets 4 or 6 (P4 and P6), due to their small volume capacity and polarity, followed by P, forming the aforementioned motifs blocking P’s critical role in position 5 or 7 (p5 and p7), making them unequivocally negative for inducing Ab production. This mainly occurred when NP and SP motifs were preceded by positively charged, long side chain (K or R) or aliphatic residues (L), as in **43436** (0701), **43270** (1502), **43218** (0405), and **43414** (1401) (HLA-DRβ1* in parenthesis); this suggested SC-BB interaction’s negative impact on PPII_L_ propensity, having negative consequences regarding the immune response. Alternately, some of these *Aotus* alleles may not have occurred in the aforementioned trials (**Figures 5**, **6**), i.e., **43436** was discarded.

Modifying physicochemical conditions led to dramatic improvement regarding elution (EL), BA and HLA-DRβ1* and Aona-DRB allele-binding ability (**Figures 5**, **6**); most modified peptides thus became strong-binders, leading to significant immunogenicity (high Ab production rate) in *Aotus* monkeys, thereby strongly contrasting with native parental peptides having poor or no BA (compare **Figure 4**) and poor or no immunological activity.

### n→ π* Electron Delocalisation in SM-COLSARSPROT Peptides

The n→ π* delocalisation effect (stabilising α_L_ and β-turns in protein structures) was also disrupted, mainly when associated with neutral polar aa N and Q (57, 58) (both having different propensities to form SC-BB H-bonds in electron interactions); it has been shown that they contribute to H-bond electron density delocalisation (59). The carbonyl oxygen donor (C=O) *n* orbital became delocalised in carbonyl BB antibonding π* orbitals (H-N), bringing both carbonyl groups close to form a single C=O···HN bond in Asn, when *i*→ *i-3* and *i*→ *i+3* interactions were formed (60, 61); *i*→ *i+2* is preponderant in β-turns and *i*→ *i+3* in α left-handed (α-_L_) helical regions.

Such interaction was analysed regarding peptides involved in B-21 and C-21 trials, mainly N and Q side chains interacting with their neighbours’ backbones in positions i−3 to i+3 (but located within the MHCII-PBR). We related this to an immune response (for the first time) and found that peptides involved in such interactions induced very low Ab titres compared to those not involved in such interactions. This suggested that a peptide having a structure with these characteristics does not adopt a PPII_L_-type extended structure in the MHCII PBR, i.e., a requirement for obtaining stable MHCII-peptide complex formation. Such peptides therefore had to be modified.

**Figure 10:**
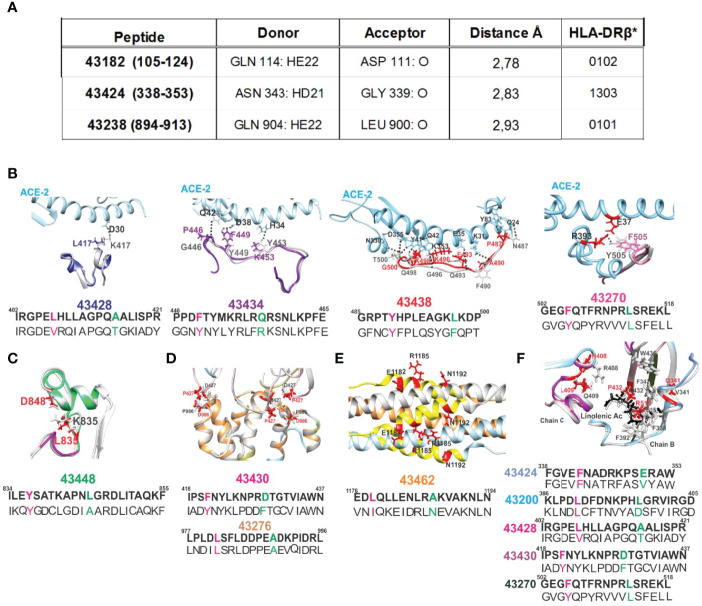
H-bonds regarding SM-COLSARSPROT peptides and ACE2 receptor (pale blue) interaction with native (grey) and modified peptides (colour). **(A)** The n→ π * delocalisation effect involved polar aa N and Q and SC-BB H-bonds forming during electron interactions. Donor and acceptor atoms are shown with Å distance from 43182, 43424, and 43238 and their corresponding HLA-DRβ1* molecules. Broken H-bonds and structural changes in interactions critical for S protein stabilisation. **(B)** The ACE2 receptor (pale blue), S protein native fragment (grey), and modified peptide minimised fragments (43428, dark blue; 43434, purple; 43438, red; and 43270, pink). Black dots show canonical ACE2 H-bonds with S protein. Replaced aa coloured as above, showing disruption of S peptide H-bond with receptor. **(C)** Modified peptide 43448 (green) location in the FPPR, showing aa involved in a salt bridge (black numbers) and replaced aa (red), indicating this salt bridge’s rupture. **(D, E)** Location of modified peptides involved in trimer, HR1, and HR2 region stabilisation and modified aa in red. **(F)** The location of aa involved in S protein hydrophobic interaction with linoleic acid (black structure). Red numbers on the structure show aa replaced in modified peptides, indicating lack of hydrophobic interactions. All images show modified aa sequences in bold letters below the native sequence; superscript numbers indicate their location in the protein.

The UCSF Chimera package (32) was used for analysing this effect regarding SM-COLSARSPROT peptides for Gln n→ π* interactions represented by H-bond formation between C=O···HN in *i* → *i-4* or *i* → *i-3*, modified peptide **43238** (894–913) where Q904 had n→ π * interaction with L900 (62). β-turn type IV_3_–derived **43424** (338–353) involved N343 having n→ π * interaction with G339 and peptide **43182** (105–124) where Q114 interacted with D111 (**Figure 10A**). All induced very low Ab titres in **B-21** and **C-21**, highlighting n→ π* delocalisation’s negative effect on the immune response.

### Modified Peptides Improved MHCII Allele Binding

Most of the 50 modified peptides had strong class II molecule BA (percentile rank 0.0 to 2.5) in **B-21** (**Figure 5**); contrasting with their native parental peptides (**Figure 4**), only one of 50 (**43242**, HLA-DRβ1*1401) modified peptides had ≥2.5 percentile purified HLA-DRβ1* EL capability (threshold level) and 11 of 50 had ≥2.5 percentile HLA-DRβ1* live homozygous cell BA (significance level). The core probability for these modified peptides to bind class II molecules (close to 1.0: data not shown) suggested a significant probability of binding predicted HLA-DRβ* or Aona-DRB alleles.

**Figure 11:**
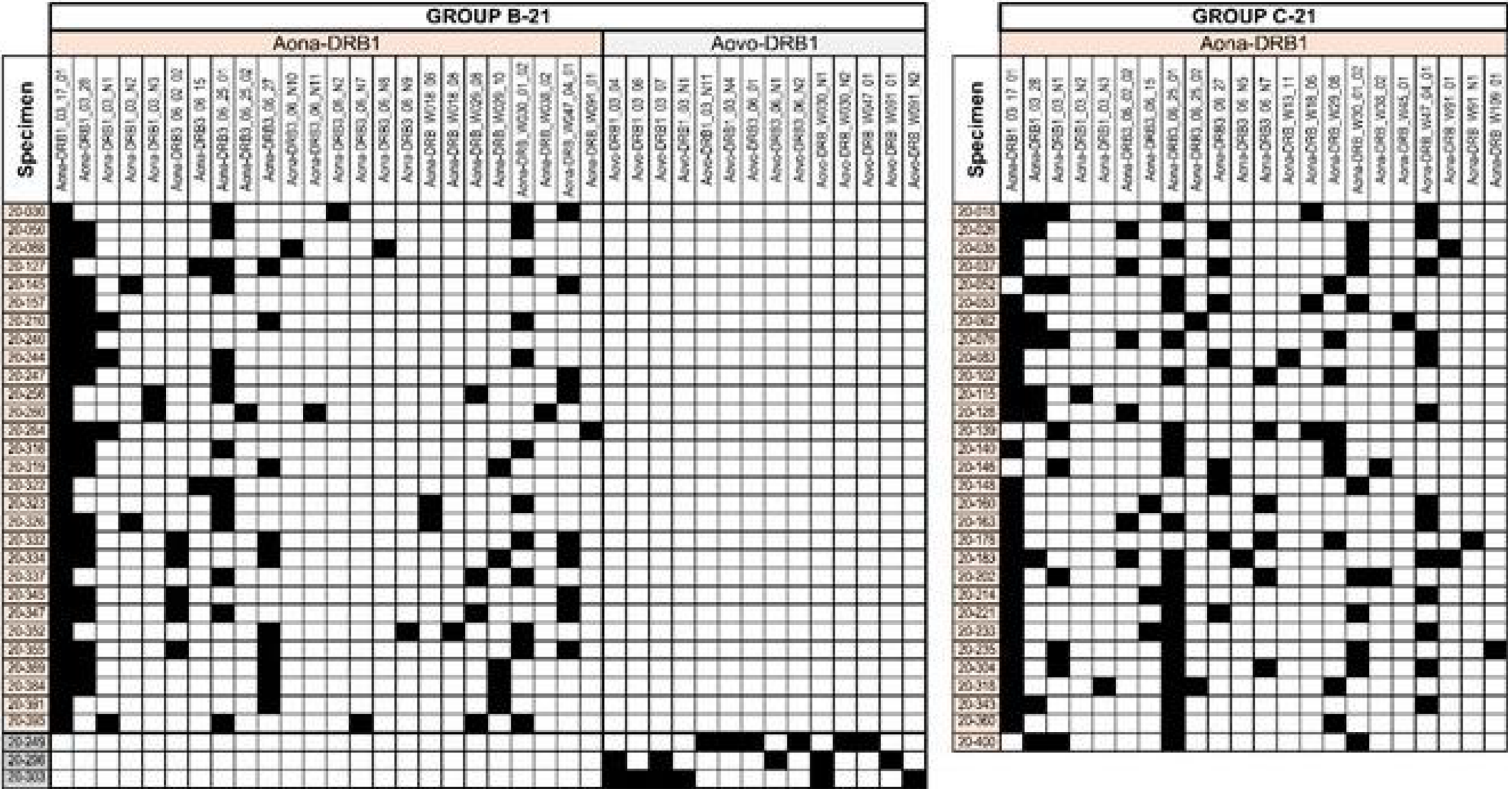
MHC-DRB typing of *Aotus* monkeys used in the trials. The Illumina NovaSeq sequencing system was used for typing the exon 2 amplicon. *A. nancymaae* monkeys’ alleles are indicated in beige, *A. vociferans* in grey. The figure indicates the amount of monkeys in which the typed alleles were detected (row) and the amount of alleles per monkey (columns) for **B-21** and **C-21**. **Figures 5–** **7** show genotyping correlated with matching HLA-DRβ1* alleles. The other DNA samples were not suitable for sequencing. The Illumina NovaSeq sequencing platform was used for typing the exon 2 amplicon. *A. nancymaae* monkey alleles are highlighted in orange and *A. vociferans* in grey; genotyping was linked with matching HLA-DR1* alleles (as shown in **Figures 5–** **7**).

NetMHCIIpan4.1’s more specific and accurately predicted EL ranking for *Aotus* allele frequency was preferred due to few Aona-DRB genotyped monkeys (~900) compared to HLA-DRβ1* (>4,000,000 people) (**Figures 5**, **6**). The server classified human HLA-DRβ1*1401, 1201, 1202, and 0802 equivalent alleles as not found (**NF**) in *Aotus* **B-21** and **C-21** because their Aona-DRB alleles did not occur in such relatively small group of monkeys or had very low frequency (some <1%). The difference between trials stressed the importance of carrying out several trials (as reported here) to identify relevant alleles having strong aa sequence SI and ID. DNA genotyping enabled accurate identification of genetic traits because up to six Aona-DRB alleles can be identified in *Aotus* monkeys (**Figure 11**), unlike two HLA-DRβ1* in humans; such difference enabled more potentially relevant SM-COLSARSPROT peptides to be recognised.

### VOC and VOI Mutating Residue Replacements

Residues having ≥1,000 VOC and VOI variations were selected from Global Initiative on Sharing Avian Influenza Database (GISAID) ‘s EpiCoV database (63); 235,111 sequences were shown to have residues having mutations (March 10^th^ 2021, when this work began) (**Figure 7**). GISAID was used for identifying aa **not** used by mutants in all SARS-CoV-2 variants and for replacing mutant residues by aa having very close or similar mass, volume, and surface but opposite polarity (**Figures 5**–**7**). This is the essence of the SUPRAMUTATIONAL concept; the virus cannot use these aa during thousands of mutations, suggesting the genetic impossibility of using them.

**Figures 3**, **5**–**7** show mutant residue replacements, serial numbers, location in a protein (in parenthesis), and the HLA-DRβ1* allele they bound to after modification. **Figure 10B** shows X-ray crystallography-determined S protein location in native peptides and residues (grey: backbone and residues) interaction with ACE2 receptor (pale blue, fuchsia, and red: backbone and residues) during invasion and the shift in orientation adopted by modified peptides where replaced residues (colour) disrupted original H-bonds (black dots) in intermolecular interaction with target receptors (UCSF Chimera package) (32).

**Figure 10B** also shows X-ray crystallography-determined native parental peptide location and that of modified peptides modelled from the structure adopted by the matching protein 3D structure fragment using UCSF Chimera (32). Immunogenic modified (bold) peptides in the receptor-binding domain (RBD) are shown, along with their location in aa sequences (small superscript numbers) and modifications (in brackets). **43428** (402–421) [L417K], **43434** (446–465) [G446P, Y449F, and Y453K], **43438** (485–500) [N487P and Q493E] and **43270** (502–518) [F515R], and **43200** (386–405) [F505F] (64). **Figures 3** and **10F** show **43424** (338–353) [V341E], **43428** (402–421) [R408H and Q409L], and **43430** (418–437) [C432T] hydrophobic interactions with linoleic acid and residues having intra-molecular interactions in the hinge region (46) or trimer stabilisation (45) during host cell membrane invasion. Helical peptides **43448** (FRPP), **43276** (trimer stabilisation), and **43462** (HR1-HR2) had intra-molecular stabilisation interactions (**Figures 10C**–**E**).

All peptide replacements compensated for the lack of antigenicity and/or poor immunogenicity due to poor HLA-DRβ1* binding or lack of it (compare **Figures 5**–**7** to **Figure 4**) and VOC and VOI region mutability, making SM-COLSARSPROT the first SUPRAMUTATIONAL, highly immunogenic peptide mixture targeting SARS-CoV-2.

### *Aotus* Immunogenetics

Sixty-one monkeys were bled for DNA genotyping; however, only 55 proved to be appropriate for DNA sequencing (29 in **B-21** and 26 in **C-21**), 26 A*. nancymaae* and 3 A*. vociferans* in **B-21**.

**Figures 5**, **6** show HLA-DRβ1* allele frequency, organised by frequency in the human population. Matching *Aotus* allele frequency (parenthesis) in these trials is shown (**Figures 5**, **6**, **8**), suggesting the best-modified peptides for inclusion. This gave **43266** (W300102) and **43418** (W3802) in HLA-DRβ1*0701; **43200**, **43272**, and **43244** in (1*031701) in HLA-DRβ1*0301’ **43180** (W2908) and **43250** (W1806) in HLA-DRβ1*1501; **43230** and **43434** (1*0328) in HLA-DRβ1*1101; **43438** (W300102) in HLA-DRβ1*0101; **43456** (W2908) in HLA-DRβ1*1302; **43174** and **43274** (W470401) in HLA-DRβ1*0401; **43232** (W2910) in HLA-DRβ1*1104; **43190** (3*0627) in HLA-DRβ1*0404; and **43224** (W2908) in HLA-DRβ1*0901. Numbers boxed in green in **Figure 8** represent peptides common to **B-21** and **C-21**, stressing these results’ reproducibility and the long-lasting memory induced by them in **B-21**. Strikingly, human alleles were found where *Aotus* allele frequency rate was high, clearly suggesting that this methodology can be used for **supramutational** peptide identification regarding SARS-CoV-2 and other diseases, i.e., a universal methodology.

Practically, all peptides having properly modified supramutational modifications were capable of inducing Ab production able to recognise native sequences in native peptides (determined by ELISA reactivity and IFA recognition in infected cells). This clearly suggested that the methodology described here promoted appropriate Ab-inducing responses, supported by the immunological results.

### SM-COLSARSPROT-Peptide CD Analysis

CD studies for ascertaining PPII_L_ propensity involved 51/53 **C-21** peptides due to the onerous task of ^1^H-NMR 3D structure determination (3) for recognising modified SM-COLSARSPROT peptides’ secondary structure elements. CD data collected at 27°C and 37°C provided striking results. **Figure 9** indicates random coil peptides, similar to ^1^H-NMR determination of highly protective, long-lived COLSARSPROT peptides (3). CD revealed that most (~85%) SM-COLSARSPROT peptides inducing high Ab titres (>0.3, 450-nm OD; **Figures 8F, G**) targeting **C-21** mixture native parental peptides had strong negative ellipticity at 190 nm (starting **below** 0 at 27°C and 37°C). Another six peptides inducing very low Ab titres had strong negative ellipticity at 190 nm (same range) (<0.2, 450-nm OD, **43420**, **43270**, and **43188** permanently and **43274**, **43430**, and **43426** before II_20_), suggesting a temperature-dependent physicochemical and immunological pattern (**Figures 8**, **9**). Four peptides had weird structures not associated with specific biological or immunological activity; eight more were insoluble.

The small local maximum around 218–220 nm (characteristic of PPII_L_ propensity) was not observed for modified peptides. Strikingly, no SM-COLSARSPROT had α-helix tendency and only three of 50 had β-turn type tendency despite native parental structure, clearly suggesting that appropriate modifications had made them firm MHCII binders and strong immunogenic anti-SARS CoV-2 variant peptides.

### Genotyping SARS-CoV-2 Variants Circulating in Colombia

The Universidad de Antioquia’s Immunovirology Group isolated SARS-CoV-2 variants in a biosafety level 3 laboratory (BSL-3). Briefly, Corpogen Laboratory (Bogotá, Colombia) used nanopore technology-based, next-generation sequencing (NGS) for identifying samples carrying SARS-CoV-2 variants following ARTIC SARS-CoV-2 sequencing protocol (65).

Variants were deposited in the GISAID database: wild-type (B.1/D614G) (EPI_ISL_536399), Alpha (EPI_ISL_4549188), Gamma (EPI_ISL_4926393), Delta (EPI_ISL_5103929), and Mu (EPI_ISL_4005445).

### Immunological Results

#### Cellular Immune Responses With SM-COLSARSPROT-Peptide Pools

T-cell responses were undetectable in all monkeys assayed. The 50-peptide mixture was only analysed for CD4^+^ T cells in group **B-21** due to the small amount of blood (1.5 ml) permitted to be drawn from small primates; 99.9% were positive responders. There were statistically significant differences between negative (*p* < 0.0001) and positive (*p* < 0.0001) controls and CD4^+^ T cells. A selected group in **C-21** (previously reported by others as cellular immunity peptide-mediators) was positive responders (2.32%) regarding CD4+ and CD8^+^ T cells and cytokines.

**Figure 12:**
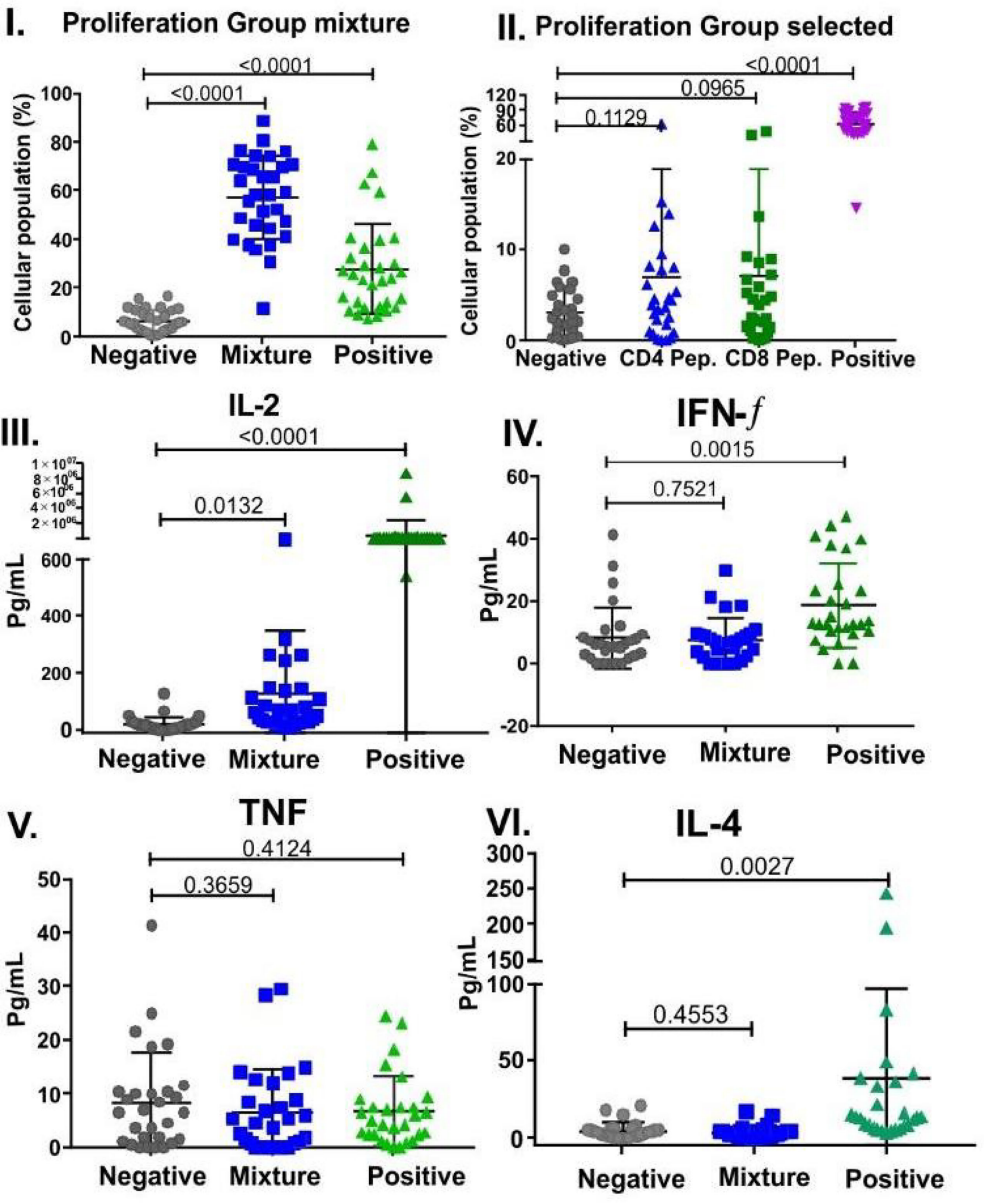
Selected peptides and cytokines’ cell proliferation in B-21 (50 peptide mixture) and C-21 trials (53 peptides). Mann–Whitney and Student’s t-tests were used for assessing statistically significant differences between negative control (culture medium) and a mixture of peptides to stimulate CD4^+^ T cells: (I). Group mixture (II). Selected peptides, showing individual *Aotus* spp. percentage CD4^+^ and CD8^+^ T cells (n = 29 for each group). CD4^+^ T cells were labelled with FITC mouse anti-*Aotus* antibody and Pacific Blue mouse anti-*Aotus* CD45RO^+^ (in-house), and CD8^+^ T cells with anti-mouse FITC-conjugated CD8^+^ T cells (RPA-T8, BD clone) and Pacific Blue mouse anti-*Aotus* CD45RO^+^. Statistically significant differences between negative control and CD4^+^ T cells, group mixture (*p* < 0.0001) and selected group (*p* = 0.1129), CD8^+^ T cells (*p* = 0.0965), and positive control (*p* < 0.0001) are shown for both groups. Cytokine-related statistically significant differences between negative control (culture medium) and a mixture of peptides for stimulating individual *Aotus* spp. CD4^+^ T cells (n=29). (III-VI) *P*-values for IL-2 (*p* = 0.0132), IFN-γ (*p* = 0.7521), TNF (*p* = 0.3659), IL-4 (*p* = 0.4553), IL-6 (*p* = 0.6502), and IL-5 (*p* = 0.3512). Individual cytokines’ positive control *p*-value compared to those for negative control: IFN-γ (*p* = 0.0015), TNF (*p* = 0.4124), IL-6 (*p* = 0.0696), IL-5 (*p* = 0.9156), IL-4 (*p* = 0.0027), and IL-2 (*p* < 0.0001).

There were statistically significant differences regarding cytokines in the 50-peptide mixture, **specifically for IL-2** (*p* = 0.0132), but *p*-values were not significant for IFN-γ (*p* = 0.7521), TNF (*p* = 0.3659), IL-6 (*p* = 0.6502), IL-5(*p* = 0.3512), or IL-4 (*p* = 0.4553). This data clearly suggested T-cell stimulation in proliferation assays and a Th1 subset activating Ab production (**Figure 12**) (66, 67).

### SM-COLSARSPROT-Related Humoral Immune Responses

#### IFA Determination

**Figure 8** shows **B-21** and **C-21** IFA reactivity on fixed slides for the aforementioned variants provided by the Universidad de Antioquia’s Immunovirology group. Reactivity increased against the wild-type (B.1/D614G) variant in 24 of 32 monkeys (75%), alpha (UK) in 22 of 32 (69%), gamma (Brazil) in 30 of 32 (94%), delta in 19 of 32 (59%) (India), and Mu (Colombia) in 21 of 31 (68%) variants 20 days after the second dose (II_20_, May 10, 2021) in **B-21** (**Figures 8A**, **B**) (32 monkeys, using Freund’s adjuvant as immunopotentiator) (**Figure 8B**). More strikingly, most maintained high IFA titres 180 days after the first dose (I_180_) or 160 after the second (II_160_, September 17/2021) as shown in **B-21**, suggesting SM-COLSARSPROT-induced long-lasting memory, i.e., why both trials are reported.

IFA reactivity increased greatly against wild-type (B.1/D614G), α, γ, δ, and μ in **C-21** (30 *Aotus* + Freund’s adjuvant). Twenty-eight of 53 (53%) peptides were common to **B-21**, 19 of 53 (36%) having 1–3 variations in their aa sequences, and 6 of 53 (~11%) new ones, not present in **B-21** (to include new VOC variations). IFA determined that 27 of 30 (90%) sera **reacted** with these variants in **C-21** 30 and 75 days after the second dose (II_30_ and II_75_ or I_105_), making the anti-SARS-CoV-2 **supramutational** mixture highly immunogenic, the best so far described against SARS-CoV-2 **VOC** and **VOI** variants.

Most of the **C-21** peptide mixture was used in **D-21**, along with the QS21 immunopotentiator (licensed for human use) used for >20 years with RTS-S ASOE recombinant malaria protein in human trials in hundreds of thousands of people. This immunopotentiator was shown to be safe with two doses in humans in 2001 and induced very high Ab titres when used with S*Pf*66 synthetic malarial vaccine (68). Immunising 42 *Aotus* led to developing ≥1:10 NAb titres against wild-type mutation (B.1/D614G) 20 days after the first immunisation in 36 of 42 blood samples analysed (85.7%) (**Figure 8C**); however, NAb titres became dramatically reduced 20 days after the second dose, suggesting QS21 immunopotentiator-associated, short-lived NAb activity in this trial, similar to the NAb levels and duration reported in other vaccine studies (69).

### ELISA Reactivity With Unmodified Native Parental Peptides

**Figure 8D** gives ELISA data showing that all 32 immunised monkeys developed Ab reacting with the 50 native parental peptides in **B-21**, i.e., wild-type (B.1/D614G) or VOC- and/or VOI-derived (small squares above peptide numbers, following established colour code, **Figures 8D, E**) 20 days after the second dose (II_20_). HLA-DRβ1*0301 (DP, KPE, PD SC-BB motifs) 0405 (KNP) had low reactivity (<0.2 absorbance at 450 nm at 1:100 sera dilution) after II_20_, which increased in II_160_. Peptides 1502 (R**N**P), 1401 (**S**PR), and 1201(APG) consistently had low Ab titres (9/50), some having SC-BB H-bond formation motifs (55, 56). Most peptides (42 of 50, 84.0%) in **B-21** induced very high Ab titres (≥0.3 absorbance at 450 nm) in monkeys having the other 26 HLA-DRβ1* alleles, suggesting this peptide mixture’s widespread immunogenicity and coverage.

Strikingly, similar to IFA titres, Ab levels remained very high in **B-21** 180 days after the first or 160 after the second (II_160_) dose; this increased in most peptides compared to II_20_ (**Figures 8D, E**). These results clearly showed that SM-COLSARSPROT induced strong, long-lasting high Ab levels against specifically modified, native, mutated aa sequences, recognising/identifying VOC and VOI aa sequences, strongly supporting SUPRAMUTATIONAL methodology.

**C-21** ELISA concerning peptides common to **B-21** (**Figures 8D–G**, green peptide numbers) confirmed this methodology’s reproducibility in the *Aotus* trials and highlighted strong reactivity specificity regarding newly VOC- and VOI-modified peptides (**Figures 8D–G**, yellow and uncoloured boxed numbers) and that such reactivity remained high 75 days after the second dose or 105 days after the first (I_105_).

It is worth stressing that SM-COLSARSPROT-induced reactivity was strong regarding specific Alpha (B.1.1.7, UK) 43216, 43276, 43246 43272, 43274, 43438, and 43456; Beta (B.1.351) 42846, 43190, 43224, 43258, 43428, 43438, and 43446; Gamma (B.1.1.28.1 or P.1) 43172, 43188, 43254, 43258, 43418, and 43428; Delta (B.1.617.2 and B.1617.1) 43264, 43266, 43272, 43274, 43418, 43434, and 43438; Epsilon (B.1.427/429) 43264, 43266, 43272, 43274, 43434, 43438, and 43446; and Mu (B.1.621) 43272 and 43274 variant mutations. This clearly suggested that a supramutational approach can deal with many genetic variations and their combinations (70).

Replacing mutant residues and inducing high Ab titres against their native, functionally relevant aa sequences (ACE2 and LA-bdg, cleavage sites S1/S2, S2’ trimer stabilisation, 6-helix bundle formation: **Figure 3**) strongly suggested that these Ab could directly block or impede (by steric hindrance) such functions, partially explaining their functional immunological activity.

**D-21** using a peptide mixture, the QS-21 immunopotentiator and most of **C-21**, involved 42 *Aotus* and showed that SM-COLSARSPROT peptides could induce NAb activity against VOC and VOI mutants targeting B.1/D614G variant (**Figure 8**), but that such activity was short-lived (<30 days).

Such highly specific Ab reactivity with the panel of modified peptide-induced native VOC and VOI variant specific peptides strongly suggested ELISA as a pertinent methodology for determining supramutational activity, better than or complementing other immunological methods like NAbs.

### Potential World Coverage by SM-COLSARSPROT Immunisation

Each peptide’s potential coverage was determined in the main ethnic groups as the SM-COLSARSPROT synthetic peptide mixture was derived from genetic VOC and VOI originally identified in China, UK, South Africa Republic (SAR), Brazil, India, USA, and Colombia, but which nowadays are extensively spread around the world. Caucasians, Hispanics, Asians, Orientals, Arabs, Blacks, and Mestizos (Colombians) have different HLA-DRβ1* allele frequencies (fuchsia in **Figures 5**, **6**, when ≥3× the world’s population), grouping 78.5% of the world’s population. SM-COLSARSPROT’s predicted minimum potential coverage is 77.6% for Asians and a maximum 83.1% for Orientals and Arabs (most being ~80%). Such coverage is quite similar to that recognised by IFA (80% to 90%) regarding wild-type (B.1/D614G) genetic variants (α, γ, δ, and μ) and ELISA titre *Aotus* sera (83.2%) reacting with peptides developed for specific HLA-DRβ1* alleles for VOC and VOI mutants and NAb targeting the Wuhan wild-type (B.1/D614G) variant (83%).

Such correlation emphasises SM-COLSARSPROT’s potential as a highly immunogenic (probably protectogenic) peptide mixture for providing desperately needed/sought-after “collective or herd immunity” against most VOC and VOI mutations with a single immunising agent: SM-COLSARSPROT synthetic peptide mixture.

## Discussion

### SM-COLSARSPROT- Immunogenicity Structural Requirements

Antigenicity- (6, 7) and immunogenicity-related structural analysis (9, 11–13, 43, 51, 52) has meticulously demonstrated that peptides must have polyproline II-like left-handed (PPII_L_) structural propensity during the first critical step: antigen presentation. In-depth physicochemical analysis of 3D structure is therefore needed to ensure that non-immunogenic peptides, non- or low-MHCII binders become immunogenic, i.e., hence the extraordinary relevance of PPII_L_ formation propensity for highly immunogenic peptide development.

PPII_L_ propensity enables peptides to establish H-bonds between specifically located peptide BB atoms and HLA-DRβ1* or Aona-DRB SC atoms to stabilise such binding (6, 7, 9, 43, 51) (**Figure 1D**), unlike secondary structures like PPI, inverse β turns, left and right α-helixes and β-turns.

Immunogenicity is thus induced *via* PPII_L_ formation being substantially restricted by local interactions, particularity side chains having adjacent carbonyls from backbone (SC-BB) n→π* interactions, represented by H-bond formation (71, 72).

Peptides’ highly immunogenic PPII_L_ formation propensity must follow clearly defined rules and constraints (6, 7, 9, 11–13, 51, 52); residues outside the PBR not involved in such interactions were only modified in −p2, −p1, p10, and p11 based on specific physicochemical rules for stabilising PPII_L_ propensity.

Often-ignored, predicted intra-peptide electron interactions also had to be modified/replaced by others involving similar mass and volume but different SC charge or polarity (**Figure 4**) to induce immunogenicity, because short polar SC residues (T>S>D>N) can induce inter- and/or intra-residue H-bonds with BB atoms (32%, 29%, 26%, and 19% frequency in proteins). They form special motifs with neighbouring P (i.e., TP, TG, SP, DG, DP, NP, PD, and DE), being α-helix and β-turn components, disturbing, and reducing PPII_L_ propensity (55, 56).

Aro residues were only allowed in specific positions to avoid Aro-Pro and Pro-Aro sequences as P was the main residue in PPII_L_ formation. Its propensity is *cis*-amide bond formation with neighbouring Aro (W>Y>F) residues, the last on the propensity scale (54, 73); however, Aro (F, Y, and W, in that order) were allowed in specific places because they were the most frequent (60%–75%) residues fitting into MHCII-PBR HLA-DRβ1* and Aona-DRB P1 (6, 7, 48, 49) (**Figures 1E**, **F**). They were modified inside the PBR due to their very strong (π-CH) interaction (53), being replaced by non-aromatic residues having the same surface and volume but opposite polarity.

Aro residue proximity of 1 to 3 residues apart was also avoided (whenever possible) because they can establish π-π face-to-face, T-shaped, parallel, or antiparallel structures (74, 75); DRβ1*15 and 16 peptides were the only exceptions, where Aro residues in P1 were also mandatory in P4 (74, 75), having dramatically negative consequences for Ab production in **43186**, **43270** (HLA-DRβ1*1502).

Several modified peptides from the same alleles were synthesised and included in SM-COLSARSPROT to maximise coverage of small HLA-DRβ1*allele differences between different ethnic groups.

### Principles for Appropriate TCR Presentation

The previously mentioned, upwardly orientated soluble side-chain rotamer orientation, volume, and charge (76) are also critical features for MHC–peptide–TCR complex formation; residues in p3 and p7 were replaced by aa having similar mass, surface, and volume by aliphatic or P aa (the latter preferred because P in these positions induced or increased PPII_L_ propensity and T-cell activation) (**Figures 1B**, **C**) (3, 77, 78).

It has also been shown the PPII_L_ structures have been more stable when preceded by negatively charged or OH-displaying residues (D, E, T, and S) in their N-terminal (in −p1 or −p2) and C-terminal regions (p10 or p11, residues), followed by positively charged residues like preferred R or K (79). Therefore, besides the nine properly orientated residues fitting into the MHCII PBR–peptide–TCR complex, the four to five extra residues in N- and C-terminal regions provided the complex with more stability, making them highly immunogenic (80, 81) and protection- or NAb-inducing. Our peptides were thus 16- to 20-mer-long rather than short 9- to 10-mer-long predicted to fit into the PBR (having very little immunogenicity).

### SARS-CoV-2 Mutability

A major problem regarding COVID-19 vaccine development concerns high SARS-CoV-2 genetic variability (reported from September 2020 onwards). This mainly occurs concerning the trimer S protein mediating viral attachment to host cells and membrane fusion (82), pentamer E viroporin-mediated ion channel formation, assembly and virus budding, and the less studied M protein interacting with S, E, and N proteins (36) (little genetic variability reported to date).

Interestingly, many mutations were located in the S protein’s functionally relevant regions (mainly in the RBD, but most in the RBM), especially those directly interacting with the ACE2 receptor, the linoleic acid binding region and the S1/S2 cleavage site; very few were located in other regions. Blocking such relevant viral functions makes them excellent targets for inducing functionally directed, immune protection-inducing efficacy.

It was quite striking that most S protein mutations (very few exceptions) occurred in regions having β-sheet or random coil secondary structures (**Figure 3**, red boxes). Our selection involved inter- and intra-molecular interactions (e.g., receptor-ligand cleavage S1/S2 sites) being mediated by β-sheet or random coil structures, whereas S and E protein surface-exposed regions having α-helix structure had different functions, i.e., membrane fusion (FP, FRPP, and HR1-HR2) and ion channel formation in E. This confirmed a clear, structural-functional dichotomy, partially explaining the tremendous immunological pressure against receptor-binding and protein-processing functions and the very efficient viral escape mechanism, as a single aa mutation knocked out a very effective immune response. The impossibility or difficulty of using some aa for SARS-CoV-2 variants at these mutation sites was quite striking, a viral disadvantage taken advantage of by our institute for this SUPRAMUTATIONAL approach.

The SM-COLSARSPROT mixture (specifically **C-21)** theoretically covers ∼80% of the world’s population; it should be stressed that our strategy was to include peptides for the human population’s main HLA-DRβ1* alleles (≥1.0% global frequency), trying to cover ~80% of it and the main ethnic groups. The **C-21** mixture can thus provide the so desperately needed “community or herd immunity”, whereas **B-21** highlighted SM synthetic peptide-induced, long-lasting memory, many being present in **C-21.**

Amazonian *Aotus* monkeys having matching human alleles were used to test the aforementioned developments. Some allele equivalences occurring with very low frequency (<1.0%) were not found in them; they were thus classified as NF because our trials involved 30–50 monkeys per trial and the probability of finding these alleles was very low (**Figures 4** and **5**, Aona-DRB column).

**Figure 13:**
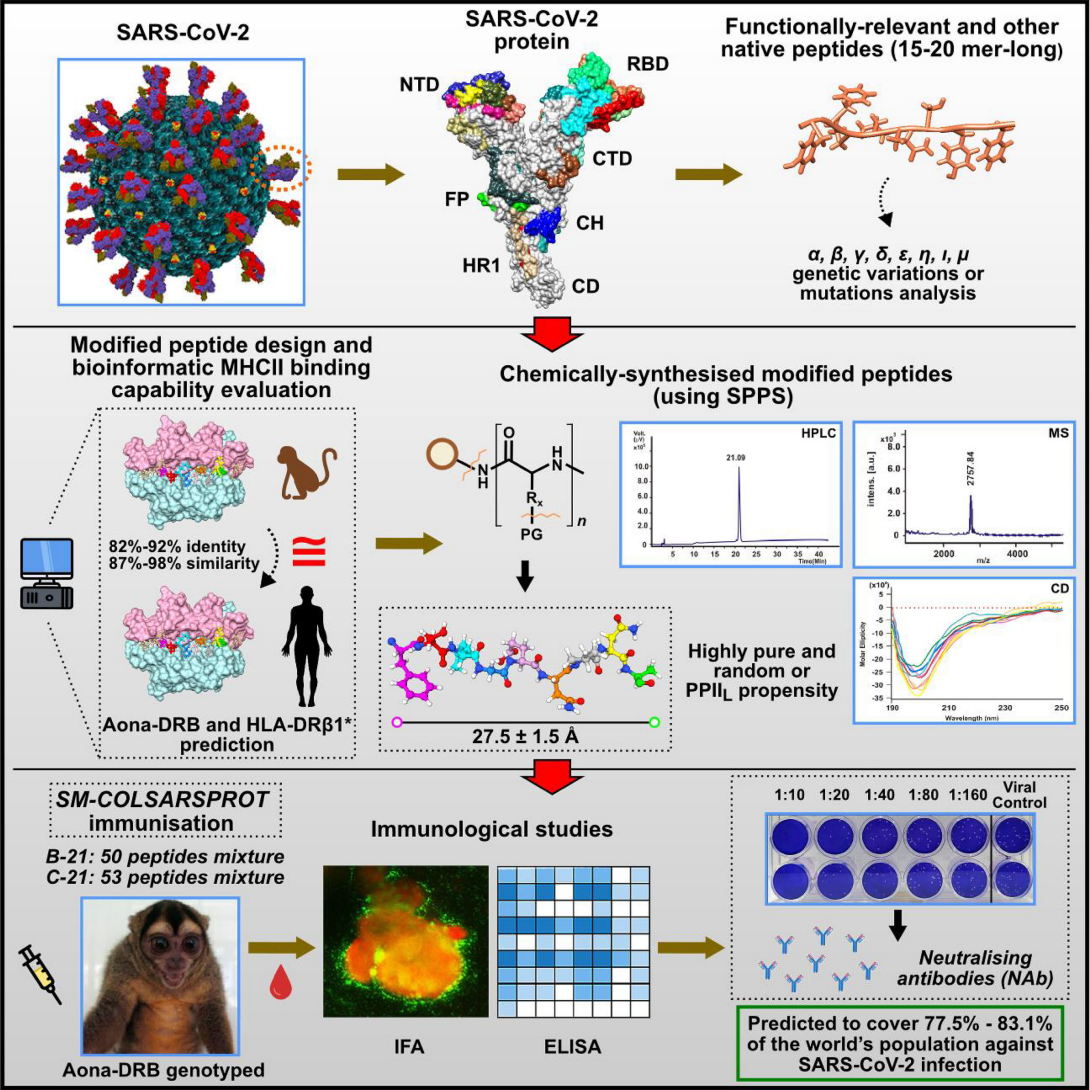
Summary of the entire process involved in obtaining SM-COLSARSPROT. Top panel: selecting functionally relevant fragments from the main SARS-CoV-2 proteins involving genetic variations. Middle panel: peptide design, production, and characterisation; Aona DRB and HLA-DRβ1* prediction. Bottom panel: immunological studies.

All these principles or rules must be considered when developing highly immunogenic, protection- or NAb-inducing peptides as antigen presentation by the MHCII–peptide–TCR complex is the first and most critical step in immune activation. **Figure 13** summarises the entire procedure used in this study, i.e., selecting the relevant fragments having genetic variations (first panel), production, characterisation, and prediction in HLA-DRB molecules (second panel) and immunological studies (third panel).

Chemically synthesised peptides are extremely pure, contaminant-free, according to the HPLC and MS, very stable at RT, do not require a cold chain for storage and transportation, and can be produced very quickly and very cheaply in not very sophisticated laboratories anywhere in the world, especially in the most needy regions. They have very high yield for immunising many people with pure, reproducible synthetic peptides (i.e., ~1,000,000 people could therefore be immunised with 10g [as produced for SPf-66 (83) if 10 micrograms of each peptide are administered per person (500 µg per dose)].

Chemists, decision-makers, ourselves, and others working in the field must assume that minimal subunit-based, multiepitope, multiprotein chemically synthesised, highly immunogenic SM-COLSARSPROT represents a tremendous advantage and solution for the currently raging threat to human health and welfare: the continuing COVID-19 pandemic. C-21 is thus an excellent example following the rules and principles expounded here, as it can be used against many other infectious diseases, including future ones, because it provides a logical, rational, and soundly established methodology for synthetic vaccine development.

## Data Availability Statement

The original contributions presented in the study are included in the article/**Supplementary Material**. Further inquiries can be directed to the corresponding author.

## Ethics Statement

The animal study was reviewed and approved by the Colombian Environmental Agency (CORPOAMAZONIA) and Fundación Instituto de Immunologia’s animal ethics committee.

## Author Contributions

MAP and MEP conceived and designed the project, analysed the data, and did the structural analysis. LP, LE, and MM did the peptide synthesis and HPLC and MS analysis. FG did the CD studies. MR, WA-J, MZ, WZ-B, and LF-Á isolated, cultured, and genotyped the SARS-CoV-2, did neutralisation assays and contributed IFA material. MA, AB, JA-C, AM-V, and CR contributed to selecting peptides and mutation analysis. CS and WA did the immunoinformatics and *Aotus* genotyping and classification with CL. YS and MF carried out immunological tests. DD did the cellular immune response studies. CA and JO did the *Aotus* experiments. JG extensively reviewed this manuscript and collaborated in experimental analysis. All authors contributed to the article and have approved the submitted version.

## Funding

This work was financed by Universidad de Ciencias Aplicadas y Ambientales (UDCA) agreement 2016-2026 and the Greenstone, International Foundation Limited, Hong Kong.

## Conflict of Interest

The authors declare that this study received funding from Greenstone International Foundation Limited. The funder was not involved in the study design and/or the collection, analysis and /or interpretation of data, the writing of the article or the decision to submit it for publication.

## Publisher’s Note

All claims expressed in this article are solely those of the authors and do not necessarily represent those of their affiliated organizations, or those of the publisher, the editors and the reviewers. Any product that may be evaluated in this article, or claim that may be made by its manufacturer, is not guaranteed or endorsed by the publisher.
