# Supplementary material for: SM-COLSARSPROT: Highly Immunogenic Supramutational Synthetic Peptides Covering the World’s Population
Source: Front Immunol. 2022 May 25;13:859905. doi: 10.3389/fimmu.2022.859905 (PMC9175637; doi:10.3389/fimmu.2022.859905)
Supplement: Supplementary file 1 [file DataSheet_1.pdf]

Allele	GB Accession
Aona-DRB_W18_06	DQ162683
Aona-DRB_W18_08	KF447760
Aona-DRB_W29_08	AY563202
Aona-DRB_W29_10	KF447743
Aona-DRB_W30_01_02	MF615354
Aona-DRB_W38_02	AY563195
Aona-DRB_W47_04_01	AY563200
Aona-DRB_W91_01	MF615347
Aona-DRB_W109_01	MF615353
Aona-DRB_W13_11	DQ162704
Aona-DRB_W18_06	DQ162683
Aona-DRB_W29_08	AY563202
Aona-DRB_W30_01_02	MF615354
Aona-DRB_W38_02	AY563195
Aona-DRB_W45_01	AY563180
Aona-DRB_W47_04_01	AY563200
Aona-DRB_W91_01	MF615347
Aona-DRB_W91_N1	OM746155
Aona-DRB1_03_17_01	AY563185
Aona-DRB1_03_28	MF615348
Aona-DRB1_03_N1	OM746129
Aona-DRB1_03_N2	OM746134
Aona-DRB1_03_N3	OM746135
Aona-DRB3_06_02_02	MF615357
Aona-DRB3_06_15	AY563213

Allele	GB Accession
Aona-DRB3_06_25_01	MF615350
Aona-DRB3_06_25_02	MF615351
Aona-DRB3_06_27	MF615352
Aona-DRB3_06_N10	OM746138
Aona-DRB3_06_N11	OM746139
Aona-DRB3_06_N2	OM746140
Aona-DRB3_06_N5	OM746143
Aona-DRB3_06_N7	OM746144
Aona-DRB3_06_N8	OM746145
Aona-DRB3_06_N9	OM746146
Aovo-DRB_W30_N1	OM746149
Aovo-DRB_W30_N2	OM746150
Aovo-DRB_W47_01	AY563227
Aovo-DRB_W91_01	MF615363
Aovo-DRB_W91_N2	OM746156
Aovo-DRB1_03_04	KF447759
Aovo-DRB1_03_06	KF447757
Aovo-DRB1_03_07	KF447758
Aovo-DRB1_03_N1	OM746157
Aovo-DRB1_03_N11	OM746162
Aovo-DRB1_03_N4	OM746159
Aovo-DRB3_06_01	KF447766
Aovo-DRB3_06_N1	OM746163
Aovo-DRB3_06_N2	OM746164
